# Effective virus-specific T-cell therapy for high-risk SARS-CoV-2 infections in hematopoietic stem cell transplant recipients: initial case studies and literature review

**DOI:** 10.1007/s11357-023-00858-7

**Published:** 2023-07-06

**Authors:** László Gopcsa, Marienn Réti, Hajnalka Andrikovics, Ilona Bobek, Gabriella Bekő, Judit Bogyó, Andrea Ceglédi, Katalin Dobos, Laura Giba-Kiss, István Jankovics, Orsolya Kis, Botond Lakatos, Dóra Mathiász, Nóra Meggyesi, Gottfried Miskolczi, Noémi Németh, Melinda Paksi, Alexandra Riczu, János Sinkó, Bálint Szabó, Anikó Szilvási, János Szlávik, Szabolcs Tasnády, Péter Reményi, István Vályi-Nagy

**Affiliations:** 1 Department of Hematology and Stem Cell Transplantation, Central Hospital of Southern-Pest, National Institute of Hematology and Infectious Diseases, 1 Nagyvárad Square, P.B. 1097, Budapest, Hungary; 2 Laboratory of Molecular Genetics, Central Hospital of Southern-Pest, National Institute of Hematology and Infectious Diseases, Budapest, Hungary; 3 Department of Intensive Care Unit, Central Hospital of Southern-Pest, National Institute of Hematology and Infectious Diseases, Budapest, Hungary; 4 Department of Central Laboratory, Central Hospital of Southern-Pest, National Institute of Hematology and Infectious Diseases, Budapest, Hungary; 5 Hungarian National Blood Transfusion Service, Karolina Út 19-21, 1113 Budapest, Hungary; 6 National Public Health and Medical Officer Service, Albert Florian Út 2-6, 1097 Budapest, Hungary; 7 Department of Infectious Diseases, Central Hospital of Southern-Pest, National Institute of Hematology and Infectious Diseases, Budapest, Hungary

**Keywords:** COVID-19, SARS-CoV-2, Virus-specific T-cells, Adoptive T-cell therapy, Hematopoietic stem cell transplantation, CliniMACS® Prodigy, Immunocompromised

## Abstract

The COVID-19 pandemic has exacerbated mortality rates among immunocompromised patients, accentuating the need for novel, targeted therapies. Transplant recipients, with their inherent immune vulnerabilities, represent a subgroup at significantly heightened risk. Current conventional therapies often demonstrate limited effectiveness in these patients, calling for innovative treatment approaches. In immunocompromised transplant recipients, several viral infections have been successfully treated by adoptive transfer of virus-specific T-cells (VST). This paper details the successful application of SARS-CoV-2-specific memory T-cell therapy, produced by an interferon-γ cytokine capture system (CliniMACS® Prodigy device), in three stem cell transplant recipients diagnosed with COVID-19 (case 1: alpha variant, cases 2 and 3: delta variants). These patients exhibited persistent SARS-CoV-2 PCR positivity accompanied by bilateral pulmonary infiltrates and demonstrated only partial response to standard treatments. Remarkably, all three patients recovered and achieved viral clearance within 3 to 9 weeks post-VST treatment. Laboratory follow-up investigations identified an increase in SARS-CoV-2-specific T-cells in two of the cases. A robust anti-SARS-CoV-2 S (S1/S2) IgG serological response was also recorded, albeit with varying titers. The induction of memory T-cells within the CD4 + compartment was confirmed, and previously elevated interleukin-6 (IL-6) and IL-8 levels normalized post-VST therapy. The treatment was well tolerated with no observed adverse effects. While the need for specialized equipment and costs associated with VST therapy present potential challenges, the limited treatment options currently available for COVID-19 within the allogeneic stem cell transplant population, combined with the risk posed by emerging SARS-CoV-2 mutations, underscore the potential of VST therapy in future clinical practice. This therapeutic approach may be particularly beneficial for elderly patients with multiple comorbidities and weakened immune systems.

**Supplementary information:**

The online version contains supplementary material available at 10.1007/s11357-023-00858-7.

## Introduction

As of May 2023, the World Health Organization (WHO) reported over 765 million confirmed cases and more than 6.9 million deaths globally due to COVID-19 [1]. Since the pandemic’s inception, the severity of COVID-19 infections among elderly, immunocompromised, and co-morbid individuals, including those undergoing immunosuppressive therapy, solid organ transplantation (SOT), or hematopoietic stem cell transplantation (HSCT), has been of significant concern [2–4]. Age-related immunosenescence may contribute significantly to the severity of COVID-19 infection and viral persistence [5]. A syndrome akin to Long COVID-19, characterized by protracted or recurrent symptoms, repeated viremia, and lung complications, is prevalent in SOT/HSCT recipients and can be accompanied by latent myocardial, renal, and neurological conditions [5].

The SARS-CoV-2 pandemic has presented a considerable challenge for patients with malignant hematological diseases and those undergoing HSCT [6, 7]. A survey by the Center for International Blood and Marrow Transplant Research (CIBMTR) reported a 30-day COVID-19 mortality rate exceeding 30% for both transplant modalities [7]. European Society for Blood and Marrow Transplantation (EBMT) data also indicated substantial mortality rates [8]. Follow-up studies concluded that COVID-19 poses a high risk to HSCT patients, especially those undergoing allogenic-HSCT and autologous HSCT within 6 months, necessitating special monitoring [2]. While initial reports indicated higher in-hospital mortality among SOT recipients, subsequent studies revealed a survival rate comparable to the general population. However, SOT recipients over 60 demonstrated a higher mortality rate and were more likely to receive treatments such as remdesivir, convalescent fresh frozen plasma (FFP), and biological therapy [9, 10].

Anti-viral and biological treatment approaches often remain ineffective; therefore, alternative therapeutic options need to be considered [11–14]. In addition to life threatening outcomes, a persistent SARS-CoV-2 infection in immunocompromised hosts also carries the risk of giving rise to series of mutations in the viral genome [15–18]. Reports of prolonged viral shedding and recurrent relapses in SOT recipients have raised concerns about the emergence of multimutation variants, possibly leading to immune escape variants [9, 19]. Drawing from experiences with other viral reactivations, the adoptive transfer of T-cell therapy was proposed to combat and clear the SARS-CoV-2 virus [20, 21]. To date, adoptive transfer of virus-specific T-cells (VST) after allogeneic HSCT or SOT has shown promise in treating several resistant or persistent viral reactivations and diseases [22]. The first successful use was reported against cytomegalovirus (CMV) reactivation/disease, Epstein-Barr virus (EBV)-associated lymphoma, resulting in complete remission in a significant proportion of patients [23, 24]. Presently, VST products for clinical use can be manufactured through ex vivo expansion or direct selection, although the former tends to be complex, time-consuming, and costly [25]. Direct selection-produced VSTs provide fresh insights into therapeutic approaches for refractory viral infections, with a major advantage being their rapid production within 12–24 h [26]. Over the past decade, an interferon-γ (IFN-γ) cytokine capture system (CCS), used via a fully automated and closed technique on the CliniMACS® Prodigy device, has been employed to treat viral infections in allogeneic HSCT settings [26].

At our center, we have successfully utilized VST treatments for viral reactivations/diseases in pediatric HSCT recipients [27, 28]. We now present the successful application of SARS-CoV-2 VST therapy using the IFN-γ CCS in the first three HSCT recipients, along with a discussion on key laboratory parameters.

## Materials and methods

### SARS-CoV-2 VST recipients

The inclusion criteria for SARS-CoV-2 VST therapy were the following: severe or critical COVID-19 in immunocompromised or HSCT recipients with specific indications for VST treatment: (i) not responding to at least 2 anti-COVID-19 therapies (anti-viral and/or biological treatment); (ii) showing persistent pulmonary infiltration, recurrent symptoms, or persistent PCR positivity (peripheral blood or nasopharyngeal swab); (iii) with at least 1 or 2 human leukocyte antigen (HLA) allele matches between recipient and donor (based on HLA-A, B, C, DR). Exclusion criteria for VST treatment were as follows: (1) absence of HLA match, (2) previous anaphylactic reaction to a blood product, (3) ongoing treatment of methylprednisolone or dexamethasone, and (4) patient unwillingness. The study was supported by the Institutional Board and approved by the Scientific and Research Ethics Committee of the Hungarian National Medical Scientific Council (ETT-TUKEB IV/2743–1/2021/EKU). For prospective data collection and analysis, all patients signed a separate informed consent form beside the EBMT/CIBMTR consent forms.

### SARS-CoV-2 VST donors

The VST donors were selected from the Hungarian National Blood Transfusion Service (OVSZ) volunteer stem cell donor system, based on their HLA-A, -B, and -DR antigen matches and COVID-19 status (either convalescent and/or vaccinated). Donors were screened by flow cytometry with SARS-CoV-2 peptide pool kit (for details, see flow cytometry methods). Donors also signed an informed consent form for participating in the procedure.

### Leukapheresis: CliniMACS® Prodigy procedure

Donors were considered eligible for donation if the virus-specific CD4 + or CD8 + T-cell percentage was > 0.01% of all CD4 + or CD8 + T-cells [27, 28]. Lymphocytes were collected by unstimulated leukapheresis performed by Spectra Optia Apheresis System and Continuous Mononuclear Cell Collection (CMNC) program (Terumo Blood and Cell Technologies Inc., Lakewood, US). A total of 1 × 10^9^ white blood cells (WBC) were used for VST separation by CliniMACS Prodigy Cytokine Capture System (IFN-gamma; CCS) System (Miltenyi Biotec, Bergisch Gladbach, Germany). This system enriches antigen specific, IFN-γ secreting CD4 + and CD8 + memory T cells in the product. The automated process started with sample preparation and antigen (MACS GMP PepTivator SARS-CoV-2 Select, reference number 170–076017, Miltenyi Biotec, Bergisch Gladbach, Germany) incubation of the cells representing the antigen-specific stimulation step. The procedure was carried out according to the manufacturer’s instructions. The required number of SARS-CoV-2-specific T-cells was immediately transfused after the flow cytometric analysis. The process time was 12 h. Excess cells were frozen with 5% DMSO using CryoMACS® Freezing Bag 50 (reference: 200-074-400) and Thermo Scientific CryoMED freezer (Model 7453) (Thermo Fisher Scientific Inc., Massachusetts, US).

### Flow cytometric analysis (FACS)

All FACS measurements were performed by BD FACS CANTO II flow cytometer and analyzed by DIVA software (BD Biosciences, San Jose, USA). Donor eligibility testing and follow-up of SARS-CoV-2 VST recipients were performed on EDTA anticoagulated whole blood. After gradient separation with Ficoll (Ficoll-Paque Plus GE Healthcare, ref.: 17-1440-02), mononuclear cells were resuspended with Human Serum Albumin-Roswell Park Memorial Institute (HSA-RPMI) and incubated with a SARS-CoV-2-specific peptide pool, called SARS-CoV-2 Select (ref: 130-127-309; Miltenyi Biotec, Bergisch Gladbach, Germany), according to the manufacturer’s instructions. The SARS-CoV-2 Select pool contains amino acids of length 9–22 from 88 peptides, 63 MHC class I-restricted and 25 MHC class II-restricted. The peptides are derived from structural proteins (spike, membrane, nucleocapsid, envelope) and non-structural proteins. For the detection of the IFN-γ, the Rapid Cytokine Inspector (CD4-CD8 T-cell) Kit (ref: 130-097-343, Miltenyi Biotec, Bergisch Gladbach, Germany), supplemented with anti-cytokine antibody RCI Anti-IFN-c-PE (ref: 130-097-600, Miltenyi Biotec Bergisch Gladbach, Germany) was used. The percentage of IFN-γ producing cells was identified within the CD4 + and CD8 + subpopulations from at least 6 × 10^5^ events.

The quality control of SARS-CoV-2 VST end product was based on the following markers: CD3, CD4, CD8, CD14, CD20 and CD45 form Miltenyi Biotec (Bergisch Gladbach, Germany). To analyze lymphoid populations of patients before and after SARS-CoV-2 VST therapy, absolute cell counts were measured using TruCount tubes of the 6-color TBNK kit (ref.: 344563, BD Biosciences, San Jose, USA). The following markers were used to measure lymphoid subpopulations: HLA-DR, CD25, TCRαβ, TCRγδ, and CD45RA and CD45RO (BD Biosciences, clone UCHL1). For the determination of T-regulatory cells, BD Human Regulatory T Cell Cocktail was used (BD Biosciences, San Jose, USA) with CD4, CD25, and CD127 staining [28].

### Measurements of multicytokine levels

HCYTA-60 K Millipore MILLIPLEX MAP-Human Cytokine/Chemokine/Growth Factor Panel A—Immunology Multiplex Assay (Merck KgaA, Darmstadt, Germany) was used to measure 20 different cytokine or chemokine levels (interferon α2, (IFNα2), IFNγ, interleukin-1α (IL-1α), IL-1β, IL-2, IL-4, IL-5, IL-6, IL-8, IL-10, IL-12, IL-13, IL-15, IL-17A, regulated upon activation, normal T-cell expressed and secreted (RANTES), monocyte chemoattractant protein-1 (MCP-1), interferonγ-induced protein 10 kDa (IP-10), macrophage-inflammatory protein-1α (MIP-1α), tumor necrosis factor α (TNFα), TNFβ) [14].

### Nasopharyngeal swab and peripheral blood SARS-CoV-2 PCR

For the detection of SARS-CoV-2, two automated equipments were used to extract RNA (EliTe InGenius® (ELITech Group, Dieren, The Netherlands) for blood samples, Seegene STARlet (Seegene Inc. Seoul, South Korea) for nasopharyngeal swabs). For RNA isolation, manufacturer-validated reagents (SP200 extraction reagent, STARMAg isolation kit, Seegene Inc, Seoul, South Korea) were used. Allplex™ SARS-CoV-2 Assay (Seegene Inc., Seoul, South Korea) was chosen for multiplex real-time PCR, due to its capability to detect 4 different SARS-CoV-2 genes simultaneously (E-gene, N-gene, RdRP gene, S-gene). PCR tests were considered negative at 40 cycle threshold (Ct) or more.

### SARS-CoV-2 serology and neutralization test

The GenScript SARS-CoV-2 Surrogate Virus Neutralization Test (sVNT) (L00847, GenScript, Piscataway, USA) was used to detect circulating neutralizing antibodies. Anti-SARS-CoV-2 virus S (S1/S2) IgG was measured with chemiluminescent immunoassay (CLIA)-quantitative assay (results expressed in antibody units, AU/ml) (Liaison XL, DiaSorin S.p.A, Saluggia, Italy). Anti-SARS-CoV-2 nucleocapside (NP) IgG was detected with chemiluminescent microparticle immunoassay (CMIA) (Abbott Laboratories, Architect, Chicago, IL, USA). The interpretation of results is determined by an index (S/CO) value, which is a ratio over threshold value. Anti-SARS-CoV-2 virus IgA was detected with enzyme-linked immunosorbent assay (ELISA) (Euroimmun, Medizinische Labordiagnostika, Lübeck, Germany, results expressed in S/CO).

### Detection of microchimerism

Genomic DNAs of recipients and donors (HSCT recipient, stem cell donor, and SARS-CoV-2 VST donor) were screened for 32 insertion-deletion polymorphisms (INDELs) [29, 30]. Chimerism testing was performed on days 1, 2, 3, 5, 7, 9, 11, 14, 21, and 28 by droplet digital PCR (ddPCR, QX200 AutoDG Droplet Digital PCR System, Bio-Rad, CA, USA) with 0.05% sensitivity.

## Results

### Case histories of the three patients

#### Case 1

A 50-year-old male patient with intermediate risk acute myeloid leukemia (AML) in measurable residual disease (MRD) negative, first complete remission (CR) underwent allogeneic-HSCT from a 10/12 (2 HLA-DPB1 antigen mismatched) matched unrelated donor with granulocyte colony-stimulating factor (G-CSF) mobilized peripheral blood stem cell (PBSC) product (Table 1). At day + 63, he developed grade 2 acute skin GVHD. On day + 70, the patient experienced sore throat without any systemic symptoms. The nasopharyngeal swab showed SARS-CoV-2 PCR positivity. The chest CT-scan was normal. The patient remained on methylprednisolone and ruxolitinib combination, and remdesivir was added. SARS-CoV-2 RNAemia was detected on day + 84; therefore, he received 3 × 2 units of COVID-19 convalescent fresh frozen plasma. Because of radiological progression, the patient was switched to baricitinib and remdesivir from day + 134. During persistent COVID-19 and in addition to poor graft function complicated by neutropenic fevers, fungal infection and multiple CMV reactivations required broad-spectrum antibiotic, posaconazol, amphotericin-B, and foscarnet treatments that were required during the hospitalization. After more than 5 months, the nasopharyngeal swab and blood PCR positivity persisted, and CT-scans showed gradual progression of bilateral ground glass opacities, consolidations, reticular, and inter-septal thickening accompanied by pleural fluid accumulation corresponding to moderate COVID-19 pneumonia. Based on the donor screening, a 4/6 HLA-matched convalescent male donor was identified. The patient received 2 doses of 5 × 10^3^/kg SARS-CoV-2 VST from the same donor on days + 222 and + 229 post-transplant.

**Table 1:** Clinical characteristics of SARS-CoV-2 VST therapy recipients

Case	1	2	3
Age, years	50	31	40
Gender	Male	Male	Male
Diagnosis	AML	Ph + B-ALL	Peripheral T-cell lymphoma
Disease state at HSCT	CR	CR	CR
HSCT	Allogeneic	Allogeneic	Autologous
Donor HLA matching	10/12 unrelated	HLA-identical sibling	NA
Conditioning regimen	Thio-Treo-Flu	TBI-Eto	TEAM
Stem cell source	PBSC	PBSC	PBSC
GVHD prophylaxis	PTCY + tacrolimus + MMF	Tacrolimus + ruxolitinib	NA
Post-HSCT course	Continuous CR	Continuous CR	At + 5-month relapse
Acute GVHD	Grade 2	Grade 2	NA
Intervals between HSCT and COVID-19	2.5 months	6 months	5 months
Treatment of COVID-19 infection	Remdesivir (5 course), dexamethasone, baricitinib, convalescent FFP	Favipiravir + bamlanivimab, remdesivir + dexamethasone convalescent FFP	Remdesivir, dexamethasone
Indication of VST therapy	Persistent COVID-19 infection with viremia and nasopharyngeal swab positivity and bilateral pneumonia	Persistent COVID-19 infection with nasopharyngeal swab positivity and bilateral pneumonia	Persistent COVID-19 infection with viremia and nasopharyngeal swab positivity and bilateral pneumonia
Intervals between COVID-19 infection and VST therapy	5 months	2.5 months	2 months
1. Dose VST administration (days from HSCT)	+ 222	+ 225	+ 218
2. Dose VST administration (days from HSCT)	+ 229	+ 240	+ 225
Peripheral blood virus clearance from VST administration	week 6	week 1	week − 3
Nasopharyngeal swab virus clearance from VST administration	week 9	week 3	week 4
Survival from COVID-19 VST	4 months	11 months	11 months
Long-term outcome	Death with GVHD associated IPS, ARDS, and MOF	Alive with extensive chronic GVHD	Alive with active peripheral T-cell lymphoma and MDS-MLD, ongoing allogeneic transplant

*VST*, virus-specific T-cell; *AML*, acute myeloid leukemia; *Ph* + *B-ALL*, Philadelphia chromosome positive acute lymphoid leukemia; *HSCT*, hematopoietic stem cell transplantation; *CR*, complete remission; *HLA*, human leukocyte antigen; *NA*, not applicable; *Thio*, thiotepa; *Treo*, treosulfan; *Flu*, fludarabine; *TBI*, total-body irradiation; *Eto*, etoposide; *TEAM*, thiotepa, etoposide, cytarabine, melphalan; *PBSC*, peripheral blood stem cells; *GVHD*, graft-versus-host disease; *PTCY*, post-transplant cyclophosphamide; *MMF*, mycofenolate-mofetil; *FFP*, fresh frozen plasma; *IPS*, interstitial pneumonia syndrome; *ARDS*, acute respiratory distress syndrome; *MOF*, multiorgan failure; *MDS-MLD*, myelodysplastic syndrome with multilineage dysplasia

#### Case 2

A 31-year-old male presented with *BCR:ABL1* positive acute lymphoid leukemia (Ph + ALL). In first CR with molecular response 5.0, he underwent allogeneic-HSCT from HLA-identical male sibling donor with G-CSF-mobilized PBSC product following with total-body irradiation (TBI) and etoposide (Table 1). On day + 150, late-onset acute grade 2 GVHD with skin and gut involvement developed. He commenced treatment with methylprednisolone and ruxolitinib, resulting in a prompt and complete resolution. On day + 177, while asymptomatic, a nasopharyngeal swab screening sample confirmed PCR positivity for SARS-CoV-2. Being at high risk, the patient received favipiravir and bamlanivimab therapy in the outpatient clinic. Eight days later, a fever developed. Remdesivir and dexamethasone treatment and 2 × 2 units of COVID-19 convalescent fresh frozen plasma were administered at the Infectious Disease Department. Prior to SARS-CoV-2 VST treatment, the chest CT scan showed bilateral interstitial pneumonia. After more than 6 weeks of persistent SARS-CoV-2 PCR positivity, the patient was invited to participate in the SARS-CoV-2 VST program. Based on the donor screening tests, a young female volunteer vaccinated twice with Pfizer-BioNTech mRNA vaccine turned out to be a 4/6 HLA-matched. On days + 225 and + 240, the patient received two doses of 5 × 10^3^/kg SARS-CoV-2 VST from the same donor. Two weeks after COVID-19 VST treatment, complete regression of pneumonia was detected by Chest CT.

#### Case 3

A 40-year-old male patient diagnosed with peripheral T-cell lymphoma underwent autologous-HSCT following a conditioning with thiotepa, cytosine-arabinoside, etoposide, and melphalan (TEAM) in MRD negative first CR (Table 1). Five months after autologous transplant, the T-cell lymphoma relapsed, and SARS-CoV-2 infection was concurrently revealed with bilateral interstitial pneumonitis on the CT-scan. The patient received dexamethasone and remdesivir treatment, which resulted in marked regression of infiltrates on CT. However, nasopharyngeal swab PCR positivity persisted, and residual pneumonia did not show further regression. Based on donor screening tests, a young 4/6 HLA-matched male convalescent donor with 2 × Gam-COVID-Vac (Sputnyik V) vaccination was found to be suitable. The patient received 2 doses of 5 × 10^4^/kg SARS-CoV-2 VST from the same donor 1 week apart on days + 218 and + 225 after autologous-HSCT.

### Characterization of the screened VST donors

Altogether, 17 donors with appropriate HLA matching and COVID-19 status for transplant recipients were investigated (3 convalescent, 12 vaccinated, and 2 combined). Although the number of cases is small, SARS-CoV-2-specific T-cells seemed to be higher in convalescent ± vaccinated donors compared to vaccinated-only donors (CD4^+^IFNγ^+^ within the T-cell gate: median 0.034%, range 0.010–0.121% versus median 0.026%, range 0.005–0.057%, *p* = 0.44; and CD8^+^IFNγ^+^ T-cells: median 0.161%, range 0.013–0.509% versus median 0.0925%, range 0.0–0.261%, *p* = 0.44) (Supplementary Table 1).

### Characteristics of leukapheresis PBMC and Prodigy system SARS-CoV-2 VST end products

Detailed composition of the leukapheresis PBMC and Prodigy SARS-CoV-2 VST negative (non-target) and positive (target) fractions and the doses of VST-cells administered are given in Supplementary Table 2. With leukapheresis, PBMC above 1 × 10^9^ was collected. The ratio of CD3 + T-cells was between 64.13 and 78.99%. The PBMC product showed CD4 + T-cell dominance (CD4 + T-cells 48.72–58.89% vs CD8 + T-cells 34.7–48.29%). In the CD4 + T-cell fraction, the proportion of IFNγ + cells was similar (0.053–0.114%). As opposed to CD4 + T-cell fraction results, the ratio of CD8 + IFNγ + cells showed wider variability (0.064–1.831%). In the target fraction of the Prodigy COVID-19 VST end product, the ratio of CD3 + T cells ranged from 67.713 to 76.345. However, changes in the ratio of T-cell subpopulations, including those showing IFNγ expression, were associated with results of SARS-CoV-2-specific T-cells measured during donor screening. The VST target fraction of the convalescent donor in case 1 contained almost identical proportions of CD4 + IFNγ + (83.71%) and CD8 + IFNγ + (84.61%) T-cells. Similar results were showed in the target fraction of COVID-19 VST product of the vaccine donor in case 2. In accordance with SARS-CoV-2-specific T-cells that are one order of magnitude smaller but balanced compared to the convalescent donor during screening, the VST target fraction contained similar proportions of CD4 + IFNγ + (79.65%) and CD8 + IFNγ + (76.39%) COVID-19 specific T-cells. The convalescent ± vaccinated donor in case 3 showed a high proportion of CD8 + IFNγ + SARS-CoV-2-specific T-cells at screening. Accordingly, the predominance of CD8 + IFNγ + (95.98%) T-cells in the VST-positive target fraction was clear compared to CD4 + IFNγ + (64%) T-cells. The predominance of CD4 + IFNγ + or CD8 + IFNγ + T-cells in the Prodigy SARS-CoV-2 VST target fraction was predicted by the proportion of SARS-CoV-2-specific CD4 + IFNγ + or CD8 + IFNγ + T-cells measured in peripheral blood at donor screening.

### Clinical course after VST administration

Following VST therapy, all three patients recovered from COVID-19 and achieved viral clearance (Table 1). No short-term side effects were observed after treatment with SARS-CoV-2 VST, including GVHD or cytokine release syndrome (CRS). However, after a few months of complete recovery from COVID-19, GVHD relapse was observed in both allogeneic transplant recipients, indicating recovery of immune functions. In terms of long-term outcome (11 months of follow-up), 2 out of 3 patients are alive. In case 1, the patient’s condition gradually improved, and CT abnormalities showed moderate regression. On day + 241, due to persistent poor graft function with 100% donor cell chimerism, he received a CD34 + cell booster with 1 × 10^6^/kg CD3^+^ T-cells from his original unrelated donor resulting improving condition and blood counts. On day + 261, the patient was discharged from hospital. The patient was checked at the outpatient clinic for 1.5 months with a gradually improving condition and blood counts. On day + 302, he developed fever and dyspnea requiring re-admission. The chest CT-scan showed bilateral, nodular, and multifocal peribronchial infiltrations. The SARS-CoV-2 PCR for nasopharyngeal swab and blood samples remained negative. Five days after the admission, the patient developed respiratory failure, requiring intubation and mechanical ventilation. Persistent fever, severe pancytopenia, increased IL-6 (189.5 pg/ml) and fibrinogen (6.4 g/l), and extremely high ferritin levels (23,960 ng/ml) developed. Causative etiopathogens could not be verified during microbiological assessment. Laboratory and clinical parameters corresponded to chronic GVHD-induced secondary hemophagocytic-lymphohistiocytosis (HLH) associated idiopathic pneumonia syndrome (IPS) with acute respiratory distress syndrome (ARDS). A combination of high-dose methylprednisolone, ruxolitinib, tocilizumab, and etanercept were ineffective. Despite ventilatory and hemodynamic supports, the patient’s clinical condition gradually deteriorated, and he died 15 days after admission. Autopsy was not performed. In case 2, the patient developed chronic extensive GVHD treated with ruxolitinib and extracorporeal photopheresis (ECP) resulting in good clinical response. Two weeks after COVID-19 VST treatment, complete regression of pneumonia was detected by chest CT. Eleven months after VST therapy, the patient’s condition is excellent, free from SARS-CoV-2, with good blood counts and his underlying disease in remission. In case 3, VST treatment resulted in complete regression of bilateral pneumonia. Eleven months after VST therapy, the patient’s condition is excellent. Peripheral T-cell lymphoma activity was detected on PET/CT-scan indicating a combination treatment with azacytidine, dexamethasone, and romidepsine. Furthermore, he developed myelodysplastic syndrome with multilineage dysplasia, therefore, becoming a candidate for an allogeneic-HSCT from his HLA-identical sibling donor.

### Laboratory findings after VST therapy

#### Characterization of peripheral blood lymphoid cell subpopulations by flow cytometry

**Fig. 1:**
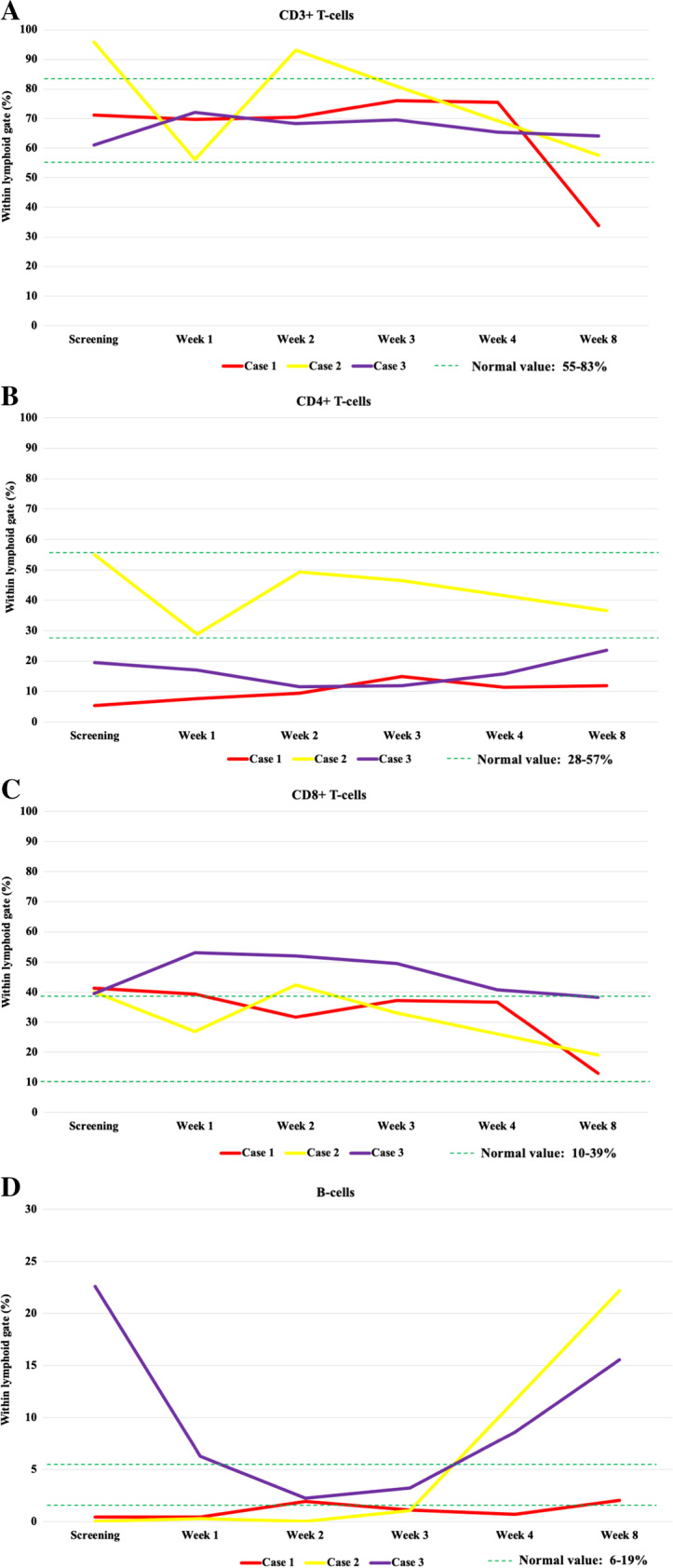

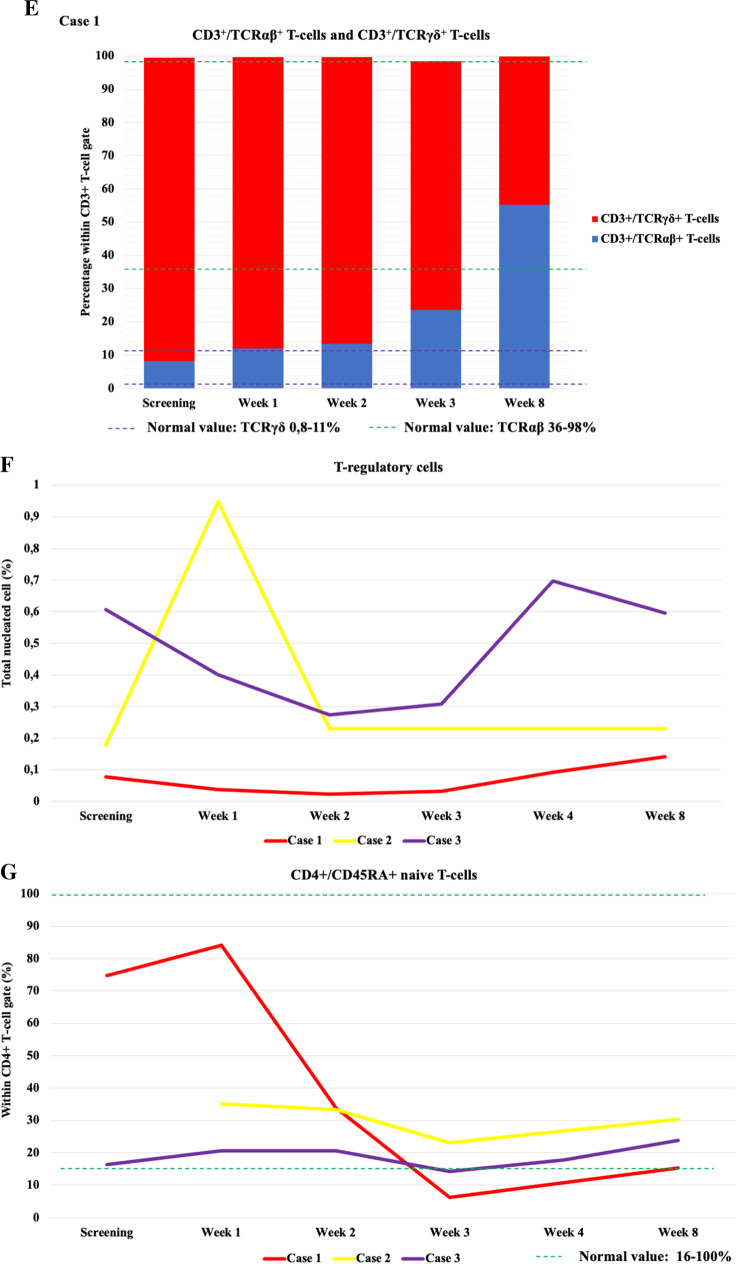

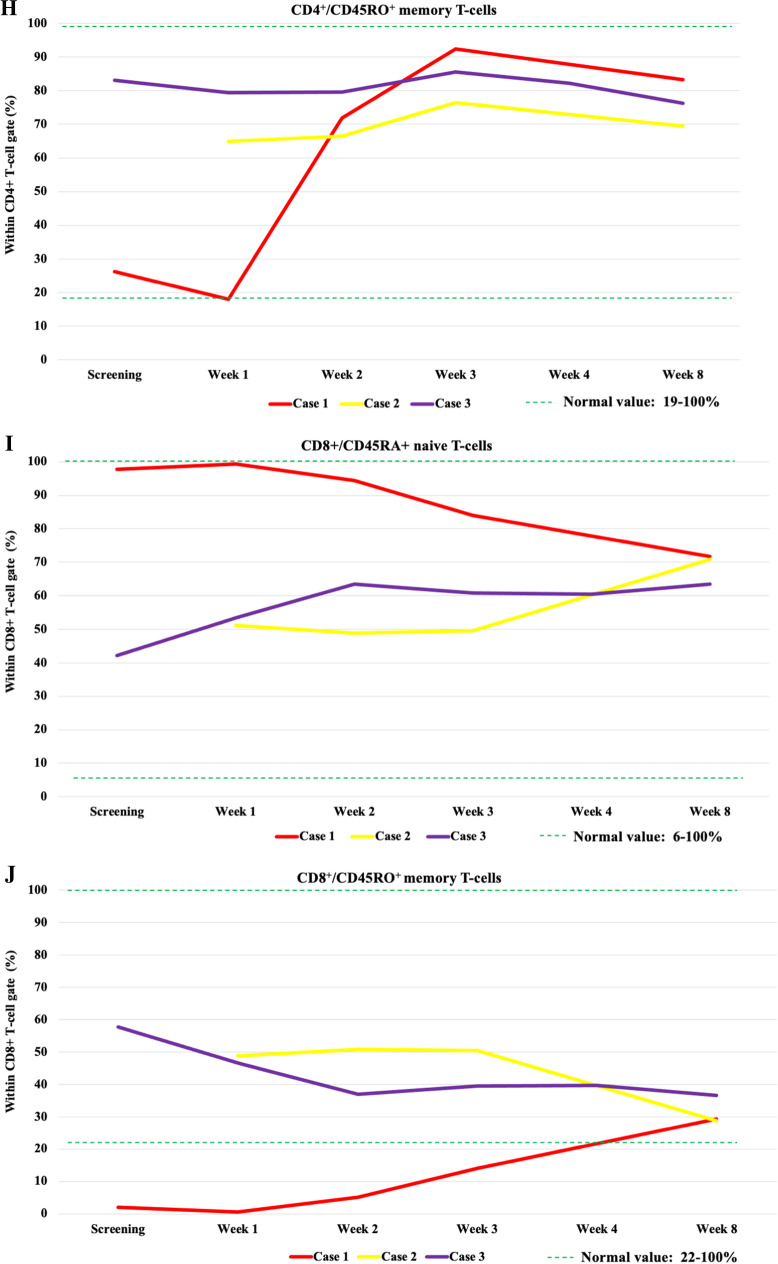
Changes in main lymphoid subpopulations by flow cytometry in 3 patients before and after COVID-19 VST treatment. **A** CD3 + T-cells. **B** CD4 + T-cells. **C** CD8 + T-cells. **D** B-cells. **E** CD3 + /TCRαβ + T-cells and CD3 + /TCRγδ + T-cells in case 1. **F** T-regulatory cells. **G** CD4 + /CD45RA + naive T-cells. **H** CD4 + /CD45RO + naive T-cells. **I** CD8 + /CD45RA + naive T-cells. **J** CD8 + /CD45RO + naive T-cells. Abbreviations: VST, virus specific T-cell; TCR, T-cell receptor

The CD3^+^, CD3^+^CD4^+^, or CD3^+^CD8^+^ T-cell ratios showed no marked change after VST therapy (Fig. 1A–C, Supplementary Table 3). VST treatment did not cause measurable changes in the major T-cell subsets. In all patients, a decrease in B cell ratio was observed at week 3 after VST administration, followed by a slow, gradual recovery in the subsequent 2 months (Fig. 1D). The increase in B cells 2 months after recovery from COVID-19 infection possibly indicates immune system regeneration. The proportion of B cells following SARS-CoV-2 virus clearance, however, showed an upward trend compared to previous values, albeit to varying degrees. In the case of the 2 allogeneic transplant recipients, the return of GVHD after COVID-19 recovery can also be considered as an indirect sign. In case 1, unlike other patients, TCRγδ + T-cell growth indicating the dominance of the innate immune system was detected during the course of COVID-19. Following VST treatment, gradual recovery and dominance of TCRαβ^+^ T-cells representing the adaptive immune system was observed in two months post-VST (Fig. 1E). In the remaining 2 patients, a dominance of TCRαβ^+^ T-cells was observed. At week 3 following VST treatment, we experienced a nadir in the naive CD4^+^CD45RA^+^ T-cell counts (Fig. 1G). After the recovery from COVID-19, a gradual increase was detected. CD4^+^CD45RO^+^ memory T-cells showed expansion in all patients by week 3, and later, their ratio stabilized (Fig. 1H). CD8^+^CD45RO^+^ compartments did not show any characteristic fluctuation after VST therapy, and after 2 months, their proportion stabilized between 30 and 40% (Fig. 1J).

#### Monitoring SARS-CoV-2 RNA by PCR in blood and nasopharyngeal swab samples

SARS-CoV-2 PCR negativity could be achieved in all three patients by 3–9 weeks after VST treatment. PCR negativity, indicating achievement of viral clearance, was defined as a Ct count above 40. Although the Ct number is a semi-quantitative method, it provides good information on the dynamics of the process. In case 1, both nasopharyngeal swab and peripheral blood specimens showed persistent PCR positivity for more than 5 months before VST therapy. By week 3 following the first dose of VST, blood samples became PCR negative. Nasopharyngeal clearance occurred by 9 weeks after VST therapy. In case 2, peripheral blood PCR positivity cleared by week 1 and nasopharyngeal swabs by week 3 after VST therapy. In case 3, blood SARS-CoV-2 PCR become negative after the antiviral treatment, but nasopharyngeal swab positivity persisted. The viral RNA clearance from nasopharyngeal swab occurred at week 4 post VST treatment (Supplementary Table 4).

#### Monitoring SARS-CoV-2-specific T-cells by flow cytometry

**Fig. 2:**
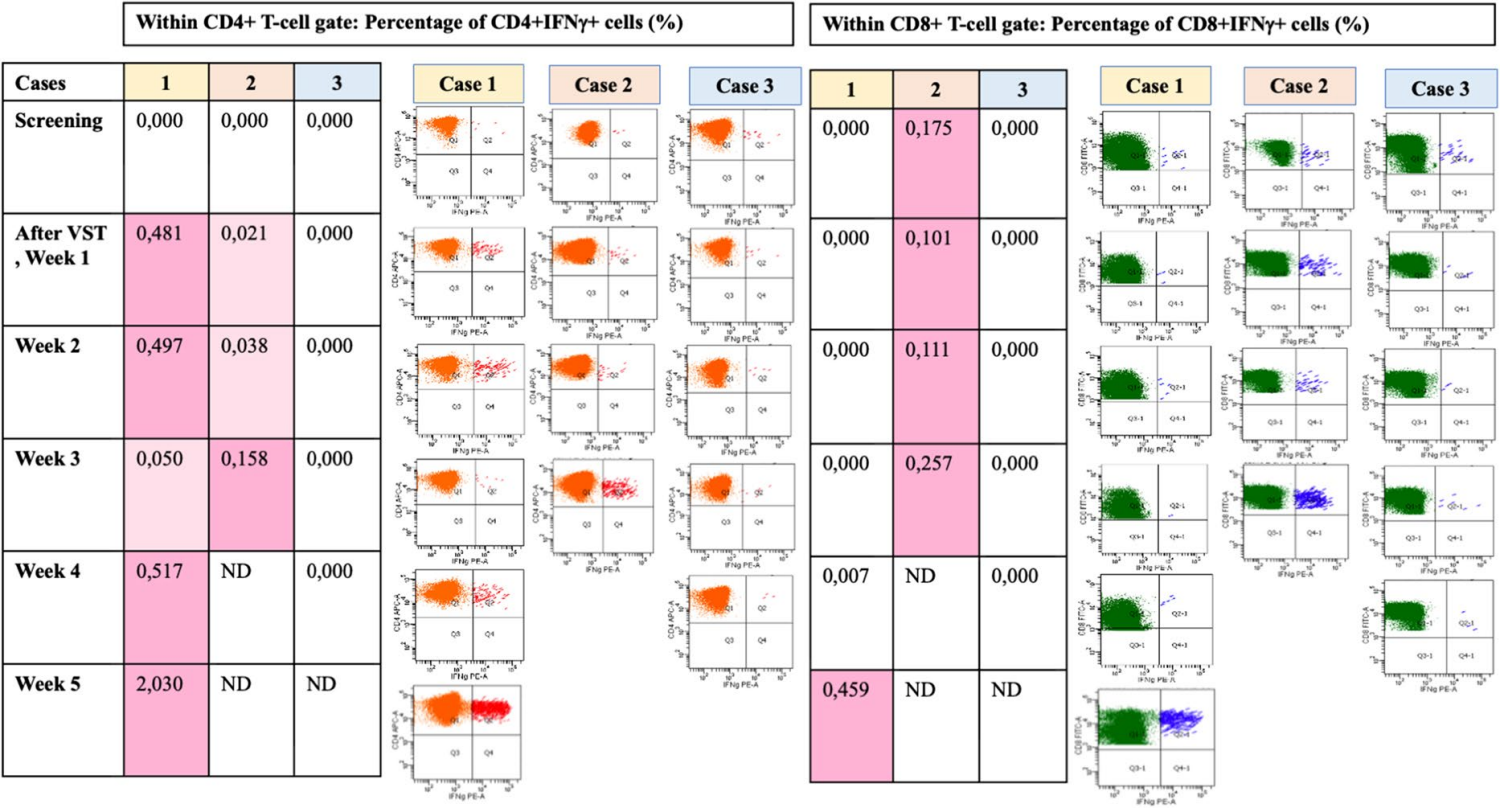
Flow cytometry analysis with Miltenyi peptide pool kit for SARS-CoV-2 virus-specific T-cells at screening and follow-up after VST therapy. Note: pale pink background: weakly positive; pink background: strongly positive. Abbreviations: VST, virus-specific T-cell; IFN-γ, interferon-γ; ND, not done

The most dramatic changes in SARS-CoV-2-specific T-cells following VST treatment were observed in case 1 (Fig. 2). During 5 months of COVID-19, SARS-CoV-2-specific T-cells were undetectable. One week after the first dose of VST, CD4^+^IFNγ^+^ SARS-CoV-2-specific T-cells became detectable (0.481%) and increased gradually. The CD8^+^IFNγ^+^ SARS-CoV-2-specific T-cells appeared much more slowly, only at week 5 (0.459% within CD8 + T-cell gate). At screening in case 2, no CD4^+^IFNγ^+^ SARS-CoV-2-specific T-cells were detected, but CD8^+^IFNγ^+^ SARS-CoV-2-specific T-cell was identified in an appropriate proportion (0.175%). At the same time, the patient was unable to clear the virus. A positive change occurred at week 3, when appropriate rates of SARS-CoV-2-specific CD4^+^IFNγ^+^ T-cells become detectable while the CD8^+^IFNγ^+^ SARS-CoV-2-specific T-cell subpopulation continued to increase. In case 3, CD4^+^ or CD8^+^IFNγ^+^ SARS-CoV-2-specific T-cells remained undetectable during screening and afterwards (Fig. 2). SARS-CoV-2 VST third-party donor cell microchimerism could not be detected in white blood cell and T-cell lineage compartments of cases 2 and 3 with 0.05% sensitivity by ddPCR method (case 1, no informative marker could be identified for microchimerism monitoring).

#### Monitoring SARS-CoV-2-specific antibody titers

**Fig. 3:**
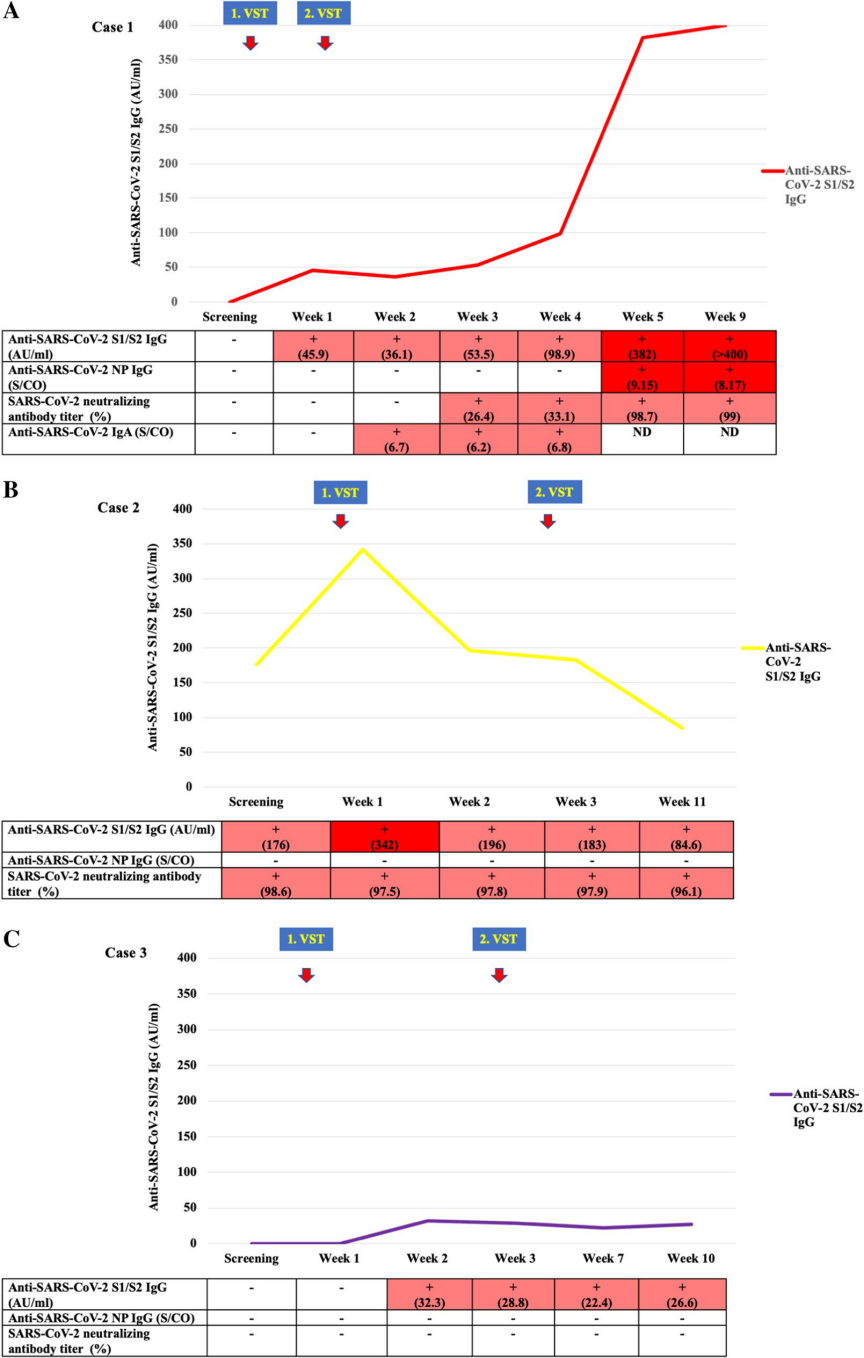
SARS-CoV-2 serology at screening and follow-up after COVID-19 VST therapy. **A** Case 1. **B** Case 2. **C** Case 3. Abbreviations: VST, virus-specific T-cell; S1/S2, spike protein; AU/ml, antibody unit/ml; IgG, immunoglobulin G; NP, nucleocapsid; S/CO, ratio over threshold value. Note: no background: negative value; pink background: positive value; red background: highly positive value

In case 1, antibody response could not be detected during screening despite a long-lasting COVID-19 (Fig. 3A). Definite antibody responses emerged following VST treatment. SARS-CoV-2 S1/S2-specific IgG was first to appear at week 1 after the VST infusion, and the titer continued to rise during the following weeks. At week 3, the titer was 53.5 AU/ml, and a measurable neutralizing antibody titer was detected. The anti-SARS CoV-2 S1/S2 IgG titer was found to be 382 AU/ml in week 5 and > 400 AU/ml in week 9. Case 2 behaved differently: anti-SARS-CoV-2 S1/S2 IgG was present even in a neutralizing antibody titer at screening, which increased following VST therapy followed by a gradual decrease (Fig. 3B). However, SARS-CoV-2 NP IgG did not appear. In case 3, all SARS-CoV-2 antibody tests were negative at the time of screening (Fig. 3C). As a result of VST treatment, the anti-SARS-CoV-2 S1/S2 IgG became positive but never reached the neutralizing titer. Furthermore, anti-SARS-CoV-2 NP IgG also did not appear.

#### Changes in multicytokine patterns

During screening and after treatment with VST, the following cytokine levels showed no increase: IFNα2, IL-1α, IL-1β, IL-2, IL-4, IL-5, IL-12, IL-13, IL-15, IL-17A, MIP-1α and TNFβ, furthermore IP-10, IL-6, IL-8, MCP-1 and RANTES levels decreased (Supplementary Table 5). In all cases, normalization of IL-6 levels was observed by week 3 after VST treatment. The kinetics of IL-8 levels were different in the 3 patients, but eventually normalized or decreased after VST treatment. In all cases, IP-10 and RANTES remained elevated, increased after SARS-CoV-2 VST therapy, and then decreased.

## Discussion

To our knowledge, this is the pioneering case series to report successful implementation of SARS-CoV-2 VST therapy, employing direct isolation through an IFN-γ CCS with the CliniMACS® Prodigy System, in adult stem cell transplant recipients diagnosed with COVID-19. Post-VST treatment, all three patients displayed regression of pulmonary infiltrates and achieved viral clearance, as demonstrated by SARS-CoV-2 PCR negativity in blood and nasopharyngeal swab samples within a span of 3–9 weeks.

Both allogeneic patients had acute GVHD prior to COVID-19 infection, which subsided due to persisting infection, and even immunosuppression treatment could be discontinued. Furthermore, after the recovery of COVID-19 infection, GVHD returned, then already in the form of a chronic process. Of course, the exact mechanism of this process is unknown, but based on recent observations, it may indeed be related to the spread of immune exhaustion, immunosenescence and SARS-CoV-2 induced senescence phenomenon. The resurgence of chronic GVHD was interpreted as an indication of the recuperation of the donor immune system post-COVID-19 infection, which could be an outcome of the CD34 + booster administered in case 1. In addition, these observations may raise the hypothesis that SARS-CoV-2-induced senescence phenomenon may increase the risk of developing many pathological process in the medium to long-term. SARS-CoV-2-specific memory T-cells were detected in both allogeneic transplant recipients. Notably, by the 5th week of VST treatment in the first case, the ratio of CD4^+^IFNγ^+^ SARS-CoV-2-specific T-cells even exceeded the numbers observed in 17 donors from our current study, as well as those reported by Ferreras et al. [31]. In case 3, no CD4^+^/CD8^+^IFNγ^+^ SARS-CoV-2-specific T-cells appeared despite VST treatment, which could be explained by the relapse of T-cell lymphoma, causing a long-term immunosuppressive state. When using VST products, cross-reactivity with other viruses cannot be ruled out, but the significance of the above is difficult to determine [32]. Clearly, SARS-CoV-2-specific humoral responses showed strong differences in all three patients. Similar humoral immune responses observed in convasecent subjects prior to the appearance of vaccines include SARS-CoV-2 anti-spike IgG levels with variable titers, often below neutralizing levels, and highly diverse anti-nucleocapsid IgG (often negative) responses. In contrast, with the proliferation of vaccines, there was often a high spike IgG response and a distinctly elevated neuralizing titer without nucleocapsid IgG. Accordingly, since patients received an adoptive T-cell transfer therapy, the humoral responses to the patch correspond to the pattern of very wide antibody responses observed among convalescent subjects. In the first case, both SARS-CoV-2 spike IgG, IgA, neutralizing titer, and NP IgG demonstrated a clear humoral response. In the second case, the SARS-CoV-2 spike IgG and neutralizing titer continued to rise under the influence of the VST, but NP IgG did not appear. Case 3, which took place in the immunosuppressive milieu of active an T-cell lymphoma, resulted in only the low titer of SARS-CoV-2 spike IgG. However, patients had a low titer of SARS-CoV-2 spike IgG response and nasopharyngeal virus clearance, which corresponds to the complete recovery. Based on this, we believe that SARS-CoV-2 VST treatment can still be considered effective. The ratio of CD4 + CD45RO + memory T-cells to VST treatment showed an expansion. In all three cases, CD4 + CD45RO + memory T-cells increased with VST treatment, but the kinetics were occasionally different. The proportion of naive T-cells CD4 + /CD45 RA + decreased with VST treatment and gradually increased between 4 and 8 weeks in all three patients. CD8 + CD45RO + memory T-cells started at different rates at screening in all three patients, and their VST kinetics also differed but stabilized between 30 and 40% in all patients 2 months after VST treatment. Regarding the cytokine levels, the normalization of IL-6 and IL-8 levels for SARS-CoV-2 VST treatment was the best indicator of recovery.

The presence of SARS-CoV-2 RNA, of course, does not prove infectivity. Unfortunately, the viability of the SARS-CoV-2 virus was not determined in the 3 patients. The development of even dangerous mutations during persistence can occur at any time in an immunocompromised patient. There is no correlation whatsoever to determine how viability is related to the emergence of further viral mutations. Therefore, whether it is a viable or a persistent SARS-CoV-2 PCR positivity with other mechanisms, it is a clear to achieve PCR negativity, which in our experience is occurred by administration of virus-specific T-cell therapy (3, 4, and 9 weeks after VST administration).

After VST treatment, we were unable to detect VST donor-derived microchimerism in white blood cells or in sorted CD3^+^ T-cells. This could be due to fact that the amount of 10^3^/kg–10^4^/kg IFNγ^+^ cells was below the detection limit of our ddPCR method (0.05%). This concept was supported by the fact that microchimerism was detected by a ddPCR with sensitivity of 0.01% in phase 1 RELEASE study using CD45RA-depleted memory T-cells in doses 1 × 10^5^–1 × 10^6^/kg produced by CliniMACS Plus® [33].

During VST therapy of CMV, adenovirus (ADV), EBV, BK, and human herpesvirus 6 (HHV-6) reactivations, based on ex vivo expansion techniques, HLA match was given great importance [26, 34, 35]. Currently, only 1 HLA allele match is required for COVID-19 T-cell therapy, whether it is CliniMACS® Prodigy or CliniMACS® Plus CD45RA methods [31, 33, 36–38]. From our institute, 9 pediatric transplant recipients treated with CMV/EBV/ADV- or multivirus-specific VST products using the CliniMACS® Prodigy CCS system were reported [27]. The treatment of viral reactivation or organ-specific viral disease proved successful in 8 instances, with 6 patients demonstrating long-term survival [27]. The effectiveness of VST treatments involving HLA disparate donors has been a subject of extensive debate [37]. In our study, we also strived to attain 4/6 HLA-matched donors. In instances of partially HLA-matching VST treatments, the response rate was observed to be between 60 and 70% [33, 34]. A 2019 review, summarizing various VST methods, reported a response rate ranging from 60 to 100% [39].

In our study, we examined not only convalescent donors but also those who had been vaccinated. It was found that an adequate cell content could also be produced from a donor who had received a vaccine. The composition of CliniMACS® Prodigy SARS-CoV-2 VST final product was closely related to the distribution of specific T-cells measured during the screening. At present, the optimal VST dose is heavily debated. In our protocol, allogeneic transplant recipients were given a dose of 5 × 10^3^ VST/kg twice over 1–2 week period, while the autologous-HSCT patient with T-cell lymphoma received an increased dose of 1 × 10^4^/kg, considering the immunological milieu of the underlying disease. The U.S. pediatric HSCT group used CliniMACS® Prodigy to treat viral reactivations [40]. With HLA-mismatched family donors, 5 × 10^3^ CD3^+^ T-cells/kg and with HLA-identical sibling donors 2.5 × 10^4^ CD3^+^ T-cells/kg VST were applied. We assume that the efficient dose of VST also depends on the production method such as ex vivo expansion, HLA-tetramer, or IFN-γ capture technique. The dose of SARS-CoV-2 VST therapy should also be guided by the proportion of non-IFNγ producing cells, which should be kept below the GVHD threshold, namely, 2 × 10^4^ non-IFNγ producing cells/kg.

With CliniMACS® Prodigy products, we do not currently know whether the CD4^+^IFNγ^+^ or CD8^+^IFNγ^+^ subpopulations were of major clinical importance. In case of cellular products manufactured by a different method, such as AlloVir® T-cell treatment (Viralym-M), it was clearly demonstrated that CD4^+^ T-cell subpopulation was of importance for the treatment of BK virus hemorrhagic cystitis [41]. The outcome of the SARS-CoV-2-specific peptide pool during donor screening could forecast which T-cell subpopulation will dominate the final positive target fraction of the SARS-CoV-2 VST product. In addition to the adoptive T-cell transfer, other therapeutic options for influencing the antiviral T-cell response are being assessed currently, such as recombinant IL-7, low-dose recombinant IL-2, Th1 activators, Th17 blockers, and immune checkpoint inhibitors [20, 21, 42].

**Fig. 4:**
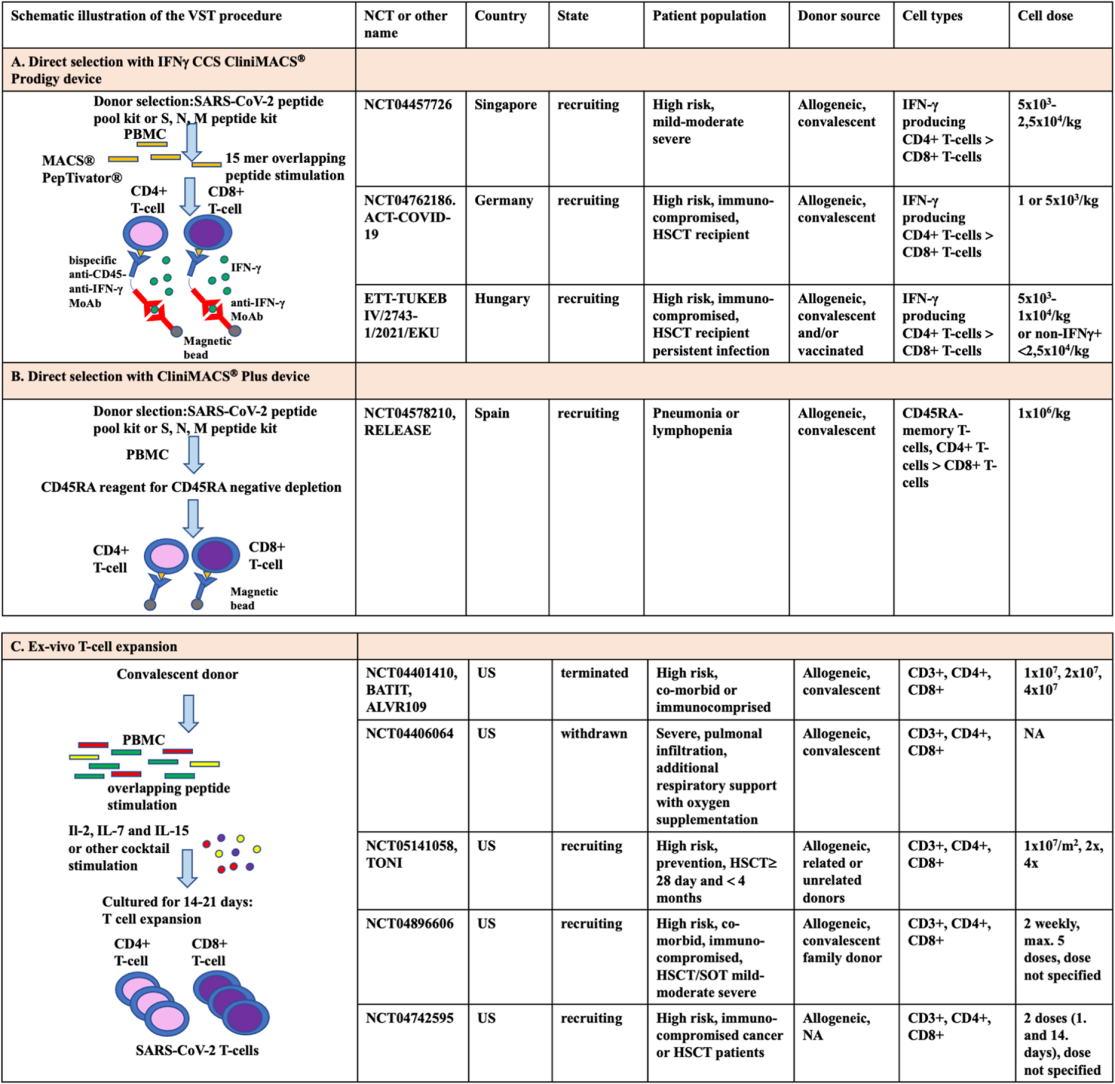

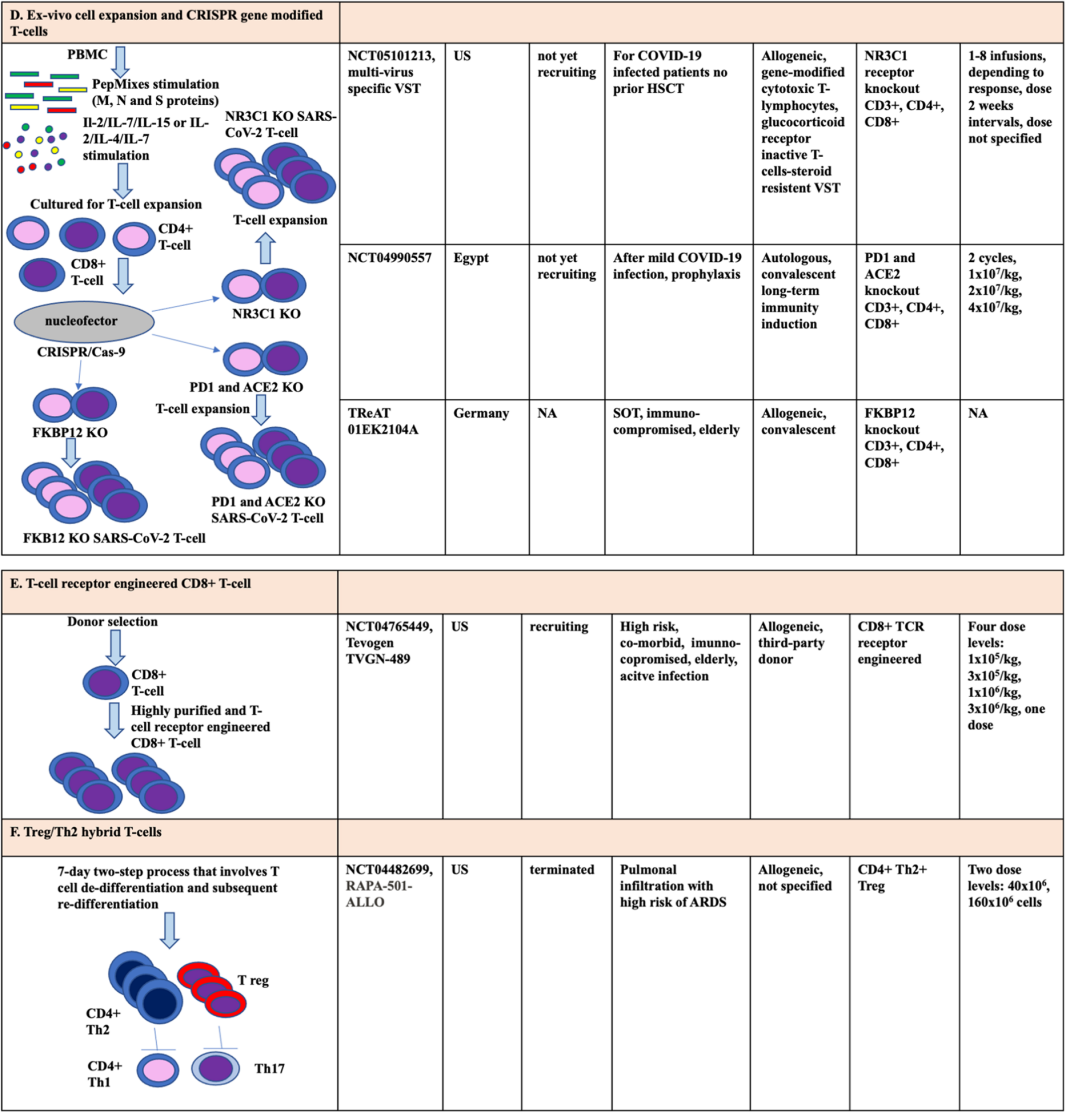
Emerging T-cell-based adoptive immunotherapy strategies to treat COVID-19 infection. Main methods: **A** direct selection with IFNγ CCS CliniMACS® Prodigy device. **B** Direct selection with CliniMACS® Plus device. **C** Ex vivo T-cell expansion. **D** Ex vivo cell expansion and CRISPR gene-modified T-cells. **E** T-cell receptor-engineered CD8 + T-cell. **F** Treg/Th2 hybrid T-cells. Abbreviations: PBMC, peripheral blood mononuclear cell; IFNγ, interferon-γ; HLA, human leukocyte antigen; Th, T helper cell; T-reg, T-regulatory cell; NCT, National Clinical Trial; DPC-OHII, Central Hospital of Southern-Pest, National Institute of Hematology and Infectious Diseases; CCS, cytokine capture system; MoAb, monoclonal antibody; ETT-TUKEB, Research Ethics Committee of the Hungarian National Medical Scientific Council; HSCT, hematopoietic stem cell transplantation; IL, interleukin; SOT, solid organ transplantation; CRISPR, RNA-controlled clustered regularly interspaced short palindromic repeats; Cas-9, caspase-9; NR3C1, nuclear receptor subfamily 3 group C member 1; PD1, programmed cell death protein1; ACE2, angiotensin-converting enzyme 2; FKBP12, FK506 binding protein 1A, 12 kDa; KO, knockout; TReAT, Tacrolimus-resistant antiviral T-cell therapy; ARDS, acute respiratory distress syndrome; TCR, T-cell receptor; NA, not available

Presently, several cellular therapy studies are underway that use allogeneic CD4 + and/or CD8 + T-cells to treat COVID-19 infection [37]. Notably, one of these trials is employing SARS-CoV-2 VSTs, produced via the IFNγ CCS with the CliniMACS® Prodigy system, mirroring the methodology used in our current report (Fig. 4A). In phase 1–2 trial, a dose of 5 × 10^3^/kg VST is used [36]. Leung et al. studied SARS-CoV-2-specific memory T-cells in 6 convalescent donors [36]. In the CD4^+^ T-cell fraction, the dominance of CD4^+^CD45RO^+^CD62L effector T-cells was found in contrast to CD8^+^ T-cells, which could be considered as another argument for the importance of CD4^+^ T-cell subgroup. Accordingly, phase 2 clinical trials, using SARS-CoV-2-specific T-cells, were launched to treat severe or high-risk COVID-19 infection (National Clinical Trial, NCT04457726, NCT04762186) [43, 44].

Beside the direct selection with IFNγCCS CliniMACS® Prodigy device, further T-cell-based adoptive immunotherapy strategies emerged to treat COVID-19 infection (see Fig. 4 for overview). The CD45RA-depleted memory T-cell infusions were developed in allogeneic-HSCT practice primarily for prophylactic use to promote immune reconstitution and prevent viral reactivations. With this approach, memory T-cells specific to pathogens encountered during the donor’s lifetime can be transferred to recipients, which can be of great importance to overcome secondary infections during COVID-19 (Fig. 4B) [31]. In CD45-depleted DLI study, the donor eligibility is the same as those for SARS-CoV-2 VST. After apheresis of the convalescent donors, CD45RA + cells undergo immunomagnetic depletion using CliniMACS® CD45RA reagent in CliniMACS® Plus system. A phase 1–2 study by a Spanish working group is ongoing (NCT04578210) investigating memory T-cell DLI from convalescent COVID-19 donors [33]. The main selection criteria are COVID-19 pneumonia and/or lymphopenia (< 1.2G/l) and O_2_ saturation ≤ 94% oxygen without need for support or ≤ 2.5 l/min through a nasal cannula. In the phase 1 RELEASE study, 9 patients were treated with 3 doses in a distribution of 3-3-3, such as 1 × 10^5^ CD45RA-T-cell/kg, 5 × 10^5^ CD45RA-T-cell/kg, and 1 × 10^6^ CD45RA-T-cell/kg [33, 37]. At 28 days, all patients recovered. The phase 2 study was conducted at a dose of 1 × 10^6^ CD45RA-T-cell/kg [33]. The above experiences provide an opportunity to combat COVID-19 infection by applying allogeneic CD45RA negative memory T-cells, which can contain the very low alloreactive T-cell content (CD45RA + T-cell content 10^2^/kg).

The SARS-CoV-2-specific T-cell infusions produced by ex vivo expansion techniques contain central memory T-cell phenotype T-cells lacking alloreactivity (Fig. 4C). The ex vivo expansion technique provides 100–200 times more cells than products manufactured by other methods, giving the opportunity to build cryopreserved biobanks [34, 45–48]. The ALVR109 off-the-shelf SARS-CoV-2 VST has been administered to 11 COVID-19-infected patients, including 5 as part of clinical trial NCT04401410 and 6 cases as part of the emergency investigational new drug FDA approval [49–51]. The first successful treatment with SARS-CoV-2 VST from ex vivo expansion technique was described in a moderately severe SARS-CoV-2 delta variant infection in an immunocompromised heart SOT patient [49]. Combined remdesivir, tocilizumab, and immunosuppressive therapies were ineffective, and persistent nasopharyngeal swab SARS-CoV-2 PCR positivity was detected [49]. The patient received off-the-shelf ALVR109 T-cell infusion from 4/8 HLA-matching donor. Three doses of ALVR109 were used, and nasopharyngeal swab virus clearance was observed already after the first dose. In addition, Vasileiou et al. administered cryopreserved ALVR109 to 4 patients, of which 3 had hematological malignancies (1 Hodgkin’s lymphoma, 1 non-Hodgkin lymphoma after autologous-HSCT, 1 chronic myeloid leukemia after allogeneic-HSCT) and 1 elderly patient with hypertension [50]. Prior to VST treatment, they were treated with steroid, convalescent FFP, and remdesivir. As a result of VST treatment, 3 patients recovered, and 1 died. Expansion of SARS-CoV-2 reactive T cells was observed in the patients. Haidar et al. gave SARS-CoV-2 VST from convalescent donors to 6 immunocompromised patients (4 lymphomas, 2 after lung transplantation). All patients showed clinical signs and viremia [51]. Viral RNA copy number was decreased. On average, 2 doses of 2 × 10^7^ cells were used. For VST treatment, 2 patients achieved a complete response, 1 had a sustained response, and 3 patients experienced a partial response followed by death. Overall, 7 out of 11 patients who received ALVR109 VST recovered, resulting in a long-term survival rate of 63% [50, 51]. Disappointingly, BATIT phase 2 study testing ALVR109 in COVID-19 infection (NCT04401410) had to be terminated early due to difficulties in selection [45]. In Viralym-M (AlloVir®), phase 2 study provided evidence that it could effectively treat BK virus hemorrhagic cystitis [41]. In addition, in a phase II clinical trial, a 92% response rate with AlloVir was demonstrated in immunocompromised patients in case of EBV, CMV, AdV, BKV, and HHV-6 infection or reactivation [34].

Another method among the anti-SARS-CoV-2 T-cell therapeutic options is the Tevogen® (TVGN)-489 T-cell product (Fig. 4E) [52]. TVGN-489 contains high-purity, T-cell receptor-engineered CD8 + T-cells developed by Tevogen Bio® Inc. The trials are currently in phase 1 trials, but several other therapeutic uses of the product are also planned [52, 53].

The RAPA-501-ALLO is also an off-the-shelf allogeneic product with a 7-day 2-step process consisting of T-cell de-differentiation and subsequent re-differentiation (Fig. 4F). The study is currently in phase 1 with severe post-intubation stage 3 COVID-19 (NCT04482699) [54]. A phase 2 study with expanded VST-cells was withdrawn by the sponsor (NCT04406064) due to the Recovery trial clearly confirming the benefit of steroids for COVID-19 [54–56]. Concomitant corticosteroids reduce the effectiveness of all VST treatments through an apoptosis-inducing effect [54, 56]. VST products could be rendered corticosteroid resistant by cell manipulation: inactivating the glucocorticoid receptor gene (nuclear receptor subfamily 3 group C member 1, NR3C1) by RNA-controlled clustered regularly interspaced short palindromic (CRISP) repeats endonuclease (Fig. 4D). A phase 1–2 (NCT05101213) clinical trial is underway in cancer patients with mild to moderate COVID-19 infection using expanded, glucocorticoid receptor modified VST [57]. In addition, studies with programmed cell death protein1 (PD1) and angiotensin-converting enzyme 2 (ACE2) knockout (inactivated by CRISPR) autologous T-cells are ongoing (NCT04990557) (Fig. 4D) [58]. Furthermore, tacrolimus-resistant FKBP12 (FK506 Binding Protein 1A, 12 kDa) knockout convalescent allogeneic SARS-CoV-2-specific T cells using CRISP-Cas9 technology is also underway to treat immunosuppressed SOT recipients [59].

In immunocompromised patients with persistent COVID-19, the emergence of multimutational SARS-CoV-2 variants is an important concern [15, 17, 60]. In a young patient with B-ALL, 12 acquired mutations were identified during 3 months of persistent SARS-CoV-2 PCR positivity [61]. Furthermore, remdesivir and monoclonal antibody-resistant mutations have been discovered in immunocompromised individuals [62, 63]. New mutations can result in the emergence of more virulent variants, as well as those with a more pronounced immune escape potential. Therefore, the use of adoptive T-cell therapy in immunocompromised individuals to reach rapid viral clearance is pivotal [64, 65].

COVID-19 has acutely highlighted the vulnerability of the elderly due to immunosenescence [66–70]. The virus has disproportionately affected older individuals, who are more likely to experience severe symptoms, hospitalization, and death. Immunosenescence is a gradual functional decline of the immune system that is associated with aging, characterized by reduced immune cell function and adaptability, leading to an increased susceptibility to infections, including SARS-CoV-2 [71]. In the elderly, the number and activity of T cells and B cells decrease, and their ability to respond to new antigens diminishes. Additionally, the functionality of innate immune cells is also compromised. This deterioration in immune function leads to increased morbidity and mortality from SARS-CoV-2 infection [66–70]. The aged immune system’s diminished capacity to mount a strong response results in less effective control and clearance of the viral infection, which can in turn lead to more extensive organ damage. The evolutionarily conserved molecular and cellular mechanisms of aging, which contribute to immunosenescence, include genomic instability, telomere attrition, epigenetic alterations, loss of proteostasis, deregulated nutrient sensing, mitochondrial dysfunction, cellular senescence, stem cell exhaustion, and altered intercellular communication [71]. Genomic instability and telomere attrition contribute to the decreased proliferation capacity of immune cells and the accumulation of dysfunctional cells; epigenetic alterations affect gene expression in immune cells, whereas loss of proteostasis can impair the function of immune proteins, leading to a suboptimal immune responses. Deregulated nutrient sensing and mitochondrial dysfunction affect the energy supply to immune cells, compromising their function. Stem cell exhaustion reduces the body’s capacity to replenish the immune system, leading to a decline in immune function over time. Finally, altered intercellular communication can disrupt the coordinated immune response to pathogens. Cellular senescence is a DNA damage-induced cellular stress response characterized by irreversible cell cycle arrest and altered cell morphology and function. Increased cellular senescence with age can lead to an accumulation of dysfunctional immune cells that not only are ineffective in mounting a response to pathogens but can also promote inflammation. Senescent cells exhibit a highly inflammatory senescence-associated secretory phenotype (SASP) characterized by the increased secretion of inflammatory mediators and factors that degrade the extracellular matrix [69]. Moreover, senescent cells can also induce senescence in neighboring cells, exacerbating inflammation. Importantly, COVID-19 associates with increased presence of senescent cells (i.e., virus-induced senescence (VIS)), which, combined with the effects of aging propel the development of hyperinflammation and, ultimately, a cytokine storm [71–73]. Collectively, these mechanisms of aging orchestrate the development of immunosenescence, reducing the effectiveness of the immune system and contributing to the increased morbidity and mortality of COVID-19 in the elderly. With respect to older HSCT recipients, numerous centers have progressively expanded the upper age limit for transplantation, now often accepting patients up to 70–75 years of age. A comparable age expansion is also observable in the case of SOT recipients. Virus-specific T-cell therapy may potentially demonstrate efficacy in these older immunocompromised patients as well, providing a promising treatment avenue for COVID-19 in this vulnerable patient population. Our understanding of the adaptive transfer of allogeneic memory T-cells in immunocompromised/HSCT/SOT recipients highlights the potential for broader clinical applications. Specifically, this method could be utilized effectively in other sub-groups of frail older adults and/or older patients with co-morbidities.

The main limitation of our study is the low number of VST-treated patients. This is primarily due to the labor-intensive, time-consuming, and expensive processes involved in establishing this specific cellular therapy. Nevertheless, we have observed that SARS-CoV-2 VST, produced via an interferon-γ cytokine capture system using the CliniMACS® Prodigy device, demonstrates promising clinical efficacy in clearing the SARS-CoV-2 virus. Both convalescent and vaccinated donors can serve as viable sources for SARS-CoV-2 VST, underscoring its versatile application. Through this therapy, we have evidenced not just clinical recovery, but also clearance of the virus itself, suggesting that adoptive T-cell transfer could present a solution for persistent SARS-CoV-2 positivity in immunocompromised hosts. As we look towards the future, the availability of SARS-CoV-2 VST therapy could become a vital instrument in decreasing the number of virus reservoirs and thereby reducing the potential for the emergence of potentially dangerous mutations. However, the expansion of SARS-CoV-2 VST therapy hinges on the outcomes of larger, more extensive clinical trials. Should these trials prove successful, and with the appropriate upscaling of capacity, this treatment could become a viable option for wider use. Of particular note is our conclusion that “off-the-shelf” SARS-CoV-2 VST could serve as a significant resource for elderly, co-morbid, or otherwise immunocompromised individuals. By overcoming immune system dysfunctions and mitigating the consequences of immunosenescence, this therapy may potentially prevent complications, promote healing, and achieve virus clearance.

### Supplementary Information

Below is the link to the electronic supplementary material.

Supplementary file1 (DOCX 23 KB)

Supplementary file2 (DOCX 23 KB)

Supplementary file3 (DOCX 25 KB)

Supplementary file4 (DOCX 20 KB)

Supplementary file5 (DOCX 22 KB)

## Data Availability

Not applicable.

## Declarations

### Disclaimer

The funders had no role in study design, data collection, data analysis, data interpretation, or writing of the report.
