# Supplementary material for: Effective virus-specific T-cell therapy for high-risk SARS-CoV-2 infections in hematopoietic stem cell transplant recipients: initial case studies and literature review
Source: GeroScience. 2023 Jul 6;46(1):1083–106. doi: 10.1007/s11357-023-00858-7 (PMC10828167; doi:10.1007/s11357-023-00858-7)
Supplement: Supplementary file 1 — Supplementary file1 (DOCX 23 KB) [file 11357_2023_858_MOESM1_ESM.docx]

**Supplementary Table 1.: Characteristics of screened SARS-CoV-2 VST donors.**

| **Donor characteristics** | **Age (years), gender** | **HLA-allele matching** | **COVID-19**  **state** | **Time interval from infection or vaccine (months)** | **Anti-SARS-CoV-2 S1/S2 IgG (AU/ml)** | **Anti-SARS-CoV-2 NP IgG (S/C)** | **Miltenyi peptide pool kit flow cytometry analysis for SARS-CoV-2 virus specific T-cells** | |
| --- | --- | --- | --- | --- | --- | --- | --- | --- |
|  |  |  |  |  | **Neutralizing antibody levels: - or %** |  | **Within CD4+ T-cell gate: % of CD4+IFNγ+** | **Within CD8+ T-cell gate: % of CD8+IFNγ+** |
| **Case 1** |  |  |  |  |  |  |  |  |
| **Donor 1** | **42, male** | **4/6** | **convalescent** | **1** | **+ (158),**  **83.3** | **+**  **(6.47)** | **0.121** | **0.161** |
| Donor 2 | 39, male | 3/6 | convalescent | 5 | + (20.5),  - | - | 0.028 | 0.217 |
| Donor 3 | 23, male | 2/6 | vaccine 2x BNT162b2 (Pfizer-BioNTech) | 2 | + (>400),  99.1 | - | 0.032 | 0.000 |
| Donor 4 | 37, male | 3/6 | vaccine 2x BNT162b2 (Pfizer-BioNTech) | 1 | + (261),  98.1 | - | 0.020 | 0.261 |
| **Case 2** |  |  |  |  |  |  |  |  |
| Donor 1 | 33, male | 10/10 (HLA-identical sibling) | convalescent | 6 | + (46.1),  - | - | 0.010 | 0.013 |
| **Donor 2** | **32, female** | **4/6** | **vaccine 2x BNT162b2 (Pfizer-BioNTech)** | **4** | **+ (>400),**  **98.9** | **-** | **0.031** | **0.049** |
| Donor 3 | 33, female | 3/6 | vaccine 2x BNT162b2 (Pfizer-BioNTech) | 3 | + (125),  88.4 | - | 0.018 | 0.024 |
| Donor 4 | 32, male | 3/6 | vaccine 2x Gam-COVID-Vac (SputnikV) | 2 | + (>400),  99.1 | - | 0.005 | 0.097 |
| **Case 3** |  |  |  |  |  |  |  |  |
| **Donor 1** | **30, male** | **4/6** | **convalescent+ vaccine 2x Gam-COVID-Vac (Sputnik V)** | **5**  **and 1** | **+ (>400),**  **99.4** | **-** | **0.034** | **0.509** |
| Donor 2 | 46, female | 4/6 | convalescent+ vaccine 1x INN-Ad26.COV2-S (Janssen) | 6  and 0.5 | + (169),  81.3 | - | 0.056 | 0.023 |
| Donor 3 | 39, male | 4/6 | vaccine 2x BNT162b2 (Pfizer-BioNTech) | 1 | + (>400),  99.0 | - | 0.038 | 0.044 |
| Donor 4 | 48, male | 4/6 | vaccine 2x Gam-COVID-Vac (Sputnik V) | 1 | + (93.2),  73 | - | 0.035 | 0.111 |
| Donor 5 | 34, male | 5/6 | vaccine 2x Gam-COVID-Vac (Sputnik V) | 1 | + (309),  89.3 | - | 0.057 | 0.132 |
| Donor 6 | 49, female | 5/6 | vaccine 2x BBIBP-CorV (Sinopharm) | 2 | + (97),  52.5 | - | 0.012 | 0.000 |
| Donor 7 | 52, male | 5/6 | vaccine 2x Gam-COVID-Vac (Sputnik V) | 1 | + (173),  88 | - | 0.015 | 0.088 |
| Donor 8 | 32, female | 5/6 | vaccine 2x Gam-COVID-Vac (Sputnik V) | 1 | + (>400),  94 | - | 0.021 | 0.098 |
| Donor 9 | 46, female | 5/6 | vaccine 2x Gam-COVID-Vac (Sputnik V) | 1 | + (>400),  99.2 | - | 0.055 | 0.112 |

Abbreviations: VST: virus specific T-cell; HLA: human leukocyte antigen; S1/S2: spike protein; IgG: immunoglobulin G; AU/ml: antibody unit/ml; NP: nucleocapside; S/CO: ratio over threshold value; IFN: interferon.
