# Supplementary material for: Effective virus-specific T-cell therapy for high-risk SARS-CoV-2 infections in hematopoietic stem cell transplant recipients: initial case studies and literature review
Source: GeroScience. 2023 Jul 6;46(1):1083–106. doi: 10.1007/s11357-023-00858-7 (PMC10828167; doi:10.1007/s11357-023-00858-7)
Supplement: Supplementary file 2 — Supplementary file2 (DOCX 23 KB) [file 11357_2023_858_MOESM2_ESM.docx]

**Supplementary Table 2.: Third-party SARS-CoV-2 virus specific T-cell product characteristics.**

|  | **Case 1** | **Case 2** | **Case 3** |
| --- | --- | --- | --- |
| **Donor** | **42-year male third-party donor** | **32-year female third-party donor** | **30-year male third-party donor** |
| **VST source** | **PBMC collected by leukapheresis** | **PBMC collected by leukapheresis** | **PBMC collected by leukapheresis** |
| **Leukapheresis product processed** | **Miltenyi Prodigy system** | **Miltenyi Prodigy system** | **Miltenyi Prodigy system** |
| **Intervals between COVID-19 infection and VST donation (months)** | **1** | **NA** | **5** |
| **Intervals between vaccine administration and VST donation (months)** | **NA** | **4** | **1** |
| **COVID-19 state** | **Convalescent** | **Vaccine** | **Convalescent+Vaccine** |
| **Leukapheresis product** |  |  |  |
| **PBMC** | **1.66x10^9^** | **1.18x10^9^** | **1.23x10^9^** |
| **CD3+ T-cells (%)** | **66.84** | **78.99** | **64.13** |
| **CD3+ T-cells (absolute number)** | **1.11x10^9^** | **0.933x10^9^** | **0.786x10^9^** |
| **CD4+ T-cells (%)** | **48.72** | **58.89** | **51.30** |
| **CD4+ T-cells (absolute number)** | **0.541x10^9^** | **0.550x10^9^** | **0.403x10^9^** |
| **CD8+ T-cells (%)** | **48.29** | **35.75** | **34.70** |
| **CD8+ T-cells (absolute number)** | **0.536x10^9^** | **0.334x10^9^** | **0.273x10^9^** |
| **CD4+IFNγ+ T-cells (%)** | **0.09** | **0.053** | **0.114** |
| **CD4+IFNγ+ T-cells (absolute number)** | **4.86x10^5^** | **2.91x10^5^** | **4.60x10^5^** |
| **CD8+IFNγ+ T-cells (%)** | **0.083** | **0.064** | **1.831** |
| **CD8+IFNγ+ T-cells (absolute number)** | **4.45x10^5^** | **2.14x10^5^** | **49.9x10^5^** |
| **Prodigy VST end product composition** |  |  |  |
| **Target (positive) fraction** |  |  |  |
| **PBMC** | **2.37x10^6^** | **3.26x10^6^** | **6.33x10^6^** |
| **CD3+ T-cells (%)** | **67.713** | **63.228** | **76.345** |
| **CD3+ T-cells (absolute number)** | **1.60x10^6^** | **2.06x10^6^** | **4.83x10^6^** |
| **CD4+ T-cells (%)** | **53.241** | **59.633** | **9.023** |
| **CD4+ T-cells (absolute number)** | **0.854x10^6^** | **1.21x10^6^** | **0.436x10^6^** |
| **CD8+ T-cells (%)** | **44.10** | **39.14** | **88.58** |
| **CD8+ T-cells (absolute number)** | **0.708x10^6^** | **0.807x10^6^** | **4.28x10^6^** |
| **CD4+IFNγ+ T-cells (absolute number)** | **0.715x10^6^** | **0.963x10^6^** | **0.279x10^6^** |
| **IFNγ+ T-cells purity within CD4+ T-cells (%)** | **83.71** | **79.65** | **64** |
| **CD8+IFNγ+ T-cells (absolute number)** | **0.599x10^6^** | **0.616x10^6^** | **4.11x10^6^** |
| **IFNγ+ T-cells purity within CD8+ T-cells (%)** | **84.61** | **76.39** | **95.98** |
| **COVID-19 VST recipient body weight (kg)** | **75** | **80** | **95** |
| **Non target (negative) fraction** |  |  |  |
| **PBMC** | **0.748x10^9^** | **0.779x10^9^** | **0.774x10^9^** |
| **CD3+ T-cells (%)** | **69.23** | **79.99** | **65.42** |
| **CD3+ T-cells (absolute number)** | **0.518x10^9^** | **0.623x10^9^** | **0.507x10^9^** |
| **CD4+ T-cells (%)** | **48.64** | **61.43** | **51.86** |
| **CD4+ T-cells (absolute number)** | **0.252x10^9^** | **0.383x10^9^** | **0.263x10^9^** |
| **CD8+ T-cells (%)** | **47.913** | **33.055** | **34.11** |
| **CD8+ T-cells (absolute number)** | **0.248x10^9^** | **0.206x10^9^** | **0.173x10^9^** |
| **CD4+IFNγ+ T-cells (%)** | **0.056** | **0.034** | **0.043** |
| **CD4+IFNγ+ T-cells (absolute number)** | **1.41x10^5^** | **1.30x10^5^** | **1.13x10^5^** |
| **CD8+IFNγ+ T-cells (%)** | **0.086** | **0.034** | **0.593** |
| **CD8+IFNγ+ T-cells (absolute number)** | **2.13x10^5^** | **0.700x10^5^** | **10.20x10^5^** |
| **End product non-IFNγ producing cells** | **3.308x10^3^/kg** | **5.454x10^3^/kg** | **3.463x10^3^/kg** |
| **End product non-IFNγ producing CD4+ cells** | **1.9x10^3^/kg** | **3.1x10^3^/kg** | **1.7x10^3^/kg** |
| **End product non-IFNγ producing CD8+ cells** | **1.4x10^3^/kg** | **2.4x10^3^/kg** | **1.8x10^3^/kg** |
| **End product IFNγ producing cells** | **17.52x10^3^/kg** | **19.738x10^3^/kg** | **46.2x10^3^/kg** |
| **End product IFNγ producing CD4+ cells** | **9.5x10^3^/kg** | **12.0x10^3^/kg** | **2.9x10^3^/kg** |
| **End product IFNγ producing CD8+ cells** | **8.0x10^3^/kg** | **7.7x10^3^/kg** | **43.3x10^3^/kg** |
| **1. VST IFNγ producing cells dose** | **5x10^3^/kg** | **5x10^3^/kg** | **1x10^4^/kg** |
| **2. VST IFNγ producing cells dose** | **5x10^3^/kg** | **5x10^3^/kg** | **1x10^4^/kg** |

Abbreviation: VST: virus specific T-cell; PBMC: peripheral blood mononuclear cells; IFN: interferon.
