# Supplementary material for: Effective virus-specific T-cell therapy for high-risk SARS-CoV-2 infections in hematopoietic stem cell transplant recipients: initial case studies and literature review
Source: GeroScience. 2023 Jul 6;46(1):1083–106. doi: 10.1007/s11357-023-00858-7 (PMC10828167; doi:10.1007/s11357-023-00858-7)
Supplement: Supplementary file 3 — Supplementary file3 (DOCX 25 KB) [file 11357_2023_858_MOESM3_ESM.docx]

**Supplementary Table 3: Flow cytometric characterization of lymphoid subpopulations at screening and after COVID-19 VST therapy.**

| **Time from VST therapy** | **Screening** | | | **Week 1** | | | **Week 2** | | | **Week 3** | | | **Week 4** | | | **Week 8** | | |
| --- | --- | --- | --- | --- | --- | --- | --- | --- | --- | --- | --- | --- | --- | --- | --- | --- | --- | --- |
| **Cases** | **1** | **2** | **3** | **1** | **2** | **3** | **1** | **2** | **3** | **1** | **2** | **3** | **1** | **2** | **3** | **1** | **2** | **3** |
| **WBC, G/l, (normal range: 4-10)** | 1.72 | 3.9 | 1.6 | 1.33 | 1.53 | 2.24 | 3.95 | 13.0 | 1.09 | 3.48 | 5.74 | 1.96 | 1.08 | ND | 2.19 | 1.94 | 10.4 | 2.27 |
| **Lymphocyte, G/l (normal range: 1,5-4)** | 0.59 | 0.68 | 0.53 | 0.26 | 0.46 | 0.41 | 0.31 | 0.86 | 0.27 | 0.24 | 0.44 | 0.25 | 0.22 | ND | 0.32 | 0.82 | 0.88 | 0.4 |
| **Percentage of lymphocyte (normal range: 20-40)** | 34.3 | 17.4 | 33.1 | 19.5 | 30.1 | 18.3 | 7.8 | 6.6 | 24.8 | 7.0 | 7.7 | 12.8 | 20.4 | ND | 14.4 | 42.3 | 8.5 | 17.6 |
| **Percentage of T-regulatory cells (% of total nucleated cells)** | 0.078 | 0.178 | 0.606 | 0.038 | 0.948 | 0.400 | 0.022 | 0.230 | 0.274 | 0.032 | ND | 0.308 | 0.092 | ND | 0.698 | 0.140 | 0.230 | 0.596 |
| **Percentage within lymphoid gate** | | | | | | | | | | | | | | | | | | |
| **Percentage of CD3+ T-cells (normal range: 55-83%)** | 71.22 | 95.78 | 61.03 | 69.73 | 56.08 | 72.09 | 70.38 | 93.09 | 68.36 | 76.01 | 80.96 | 68.48 | 75.47 | ND | 65.37 | 33.80 | 57.50 | 64.16 |
| **Percentage of CD3+/CD8+ T-cells (normal range: 10-39%)** | 41.29 | 40.17 | 39.56 | 39.31 | 26.82 | 53.16 | 31.64 | 42.36 | 52.04 | 37.16 | 33.18 | 49.52 | 36.66 | ND | 40.72 | 12.96 | 19.13 | 38.33 |
| **Percentage of CD3+/CD4+ T-cells (normal range: 28-57%)** | 5.33 | 54.93 | 19.45 | 7.72 | 28.80 | 17.12 | 9.39 | 49.35 | 11.66 | 14.97 | 46.53 | 11.98 | 11.35 | ND | 15.86 | 11.84 | 36.67 | 23.64 |
| **Percentage of CD3-/CD16+56+ NK-cells (normal range: 7-31%)** | 27.21 | 3.84 | 15.35 | 2.53 | 42.64 | 19.37 | 24.36 | 6.58 | 26.25 | 2.72 | 17.52 | 24.70 | 1.79 | ND | 20.62 | 63.98 | 19.61 | 19.73 |
| **Percentage of CD19+ B-cells (normal range: 6-19%)** | 0.47 | 0.03 | 22.62 | 0.46 | 0.28 | 6.28 | 1.96 | 0.05 | 2.26 | 1.1 | 1.09 | 3.22 | 0.69 | ND | 8.61 | 2.03 | 22.22 | 15.56 |
| **Percentage within CD3+ T-cell gate** | | | | | | | | | | | | | | | | | | |
| **Percentage of CD3+/TCRαβ+ T-cells (normal range: 36-98%)** | 8.1 | ND | 98.50 | 11.9 | 99.10 | 98.60 | 13.4 | 99.20 | 98.50 | 23.5 | 98.90 | 98.10 | ND | ND | 98.80 | 55.26 | 98.98 | 98.60 |
| **Percentage of CD3+/TCRγδ+ T-cells (normal range: 0,8-11%)** | 91.4 | ND | 1.50 | 87.8 | 0.90 | 1.40 | 86.4 | 0.80 | 1.40 | 75.1 | 1.10 | 1.90 | ND | ND | 1.20 | 44.58 | 0.87 | 1.40 |
| **Percentage of CD3+/CD25+ T-cells (normal range: >1%)** | ND | ND | ND | ND | 1.50 | 2.80 | ND | 28.30 | 1.90 | ND | 29.80 | 3.50 | ND | ND | 8.60 | 9.71 | 7.89 | 7.10 |
| **Percentage of CD3+/HLA-DR+ T-cells (normal range: 2-12%)** | ND | ND | ND | ND | 12.30 | 25.20 | ND | 25.90 | 9.90 | ND | 19.90 | 24.30 | ND | ND | 28.20 | 5.96 | 13.53 | 32.20 |
| **Percentage within CD4+ T-cell gate** | | | | | | | | | | | | | | | | | | |
| **Percentage of CD4+/CD45RA+ naive T-cells (normal range: 16-100%)** | 74.8 | ND | 16.40 | 84.1 | 35.10 | 20.60 | 33.9 | 33.40 | 20.60 | 6.3 | 23.20 | 14.20 | ND | ND | 17.70 | 15.34 | 30.37 | 23.90 |
| **Percentage of CD4+/CD45RO+ memory T-cells (normal range: 19-100%)** | 26.2 | ND | 83.20 | 17.9 | 65.00 | 79.40 | 71.9 | 66.50 | 79.60 | 92.5 | 76.50 | 85.60 | ND | ND | 82.20 | 83.28 | 69.41 | 76.30 |
| **Percentage within CD8+ T-cell gate** | | | | | | | | | | | | | | | | | | |
| **Percentage of CD8+/CD45RA+ naive T-cells (normal range: 6-100%)** | 97.8 | ND | 42.10 | 99.4 | 51.10 | 53.50 | 94.4 | 48.80 | 63.50 | 84.1 | 49.60 | 60.80 | ND | ND | 60.50 | 71.64 | 70.76 | 63.50 |
| **Percentage of CD8+/CD45RO+ memory T-cells (normal range: 22-100%)** | 2.0 | ND | 57.70 | 0.6 | 48.80 | 46.60 | 5.1 | 50.90 | 36.90 | 14.1 | 50.40 | 39.50 | ND | ND | 39.70 | 29.27 | 28.73 | 36.70 |

Abbreviations: WBC: white blood cells; ND: not done.
