# Supplementary material for: Effective virus-specific T-cell therapy for high-risk SARS-CoV-2 infections in hematopoietic stem cell transplant recipients: initial case studies and literature review
Source: GeroScience. 2023 Jul 6;46(1):1083–106. doi: 10.1007/s11357-023-00858-7 (PMC10828167; doi:10.1007/s11357-023-00858-7)
Supplement: Supplementary file 4 — Supplementary file4 (DOCX 20 KB) [file 11357_2023_858_MOESM4_ESM.docx]

Supplementary Table 4: Nasopharyngeal swab and peripheral blood SARS-CoV-2 PCR results before and follow-up after COVID-19 VST therapy.

**Case 1**

| **Weeks from COVID-19 VST** | **Nasopharyngeal swab SARS-CoV-2 virus Real-time PCR** | | | **Peripheral blood SARS-CoV-2 virus Real-time PCR** | | |
| --- | --- | --- | --- | --- | --- | --- |
| **COVID-19 genes PCR Ct number, interpretation** | **E** | **Rdrp/S** | **N** | **E** | **Rdrp/S** | **N** |
| Month -5 | **NA (+)** | **NA (+)** | **NA (+)** | **36.77 (+)** | **36.66 (+)** | **>40 (-)** |
| Month -4 | **18.93 (+)** | **18.57 (+)** | **19.16 (+)** | **33.79 (+)** | **33.96 (+)** | **35.09 (+)** |
| Screening | **19.91 (+)** | **19.42 (+)** | **19.84 (+)** | **NA (+)** | **NA (+)** | **NA (+)** |
| Week 1 | **18.92 (+)** | **18.48 (+)** | **18.46 (+)** | **32.11 (+)** | **32.39 (+)** | **33.22 (+)** |
| Week 2 | **24.14 (+)** | **23.14 (+)** | **24.02 (+)** | **38.53 (+)** | **36.43 (+)** | **36.32 (+)** |
| Week 3 | **19.51 (+)** | **18.42 (+)** | **18.72 (+)** | **>40 (-)** | **>40 (-)** | **>40 (-)** |
| Week 4 | **20.17 (+)** | **19.59 (+)** | **20.53 (+)** | **>40 (-)** | **39.56 (+)** | **>40 (-)** |
| Week 5 | **21.56 (+)** | **20.86 (+)** | **20.49 (+)** | **ND** | | |
| Week 6 | **ND** | | | **>40 (-)** | **>40 (-)** | **>40 (-)** |
| Week 7 | **NA (+)** | **NA (+)** | **NA (+)** | **ND** | | |
| Week 8 | **31.7 (+)** | **31.0 (+)** | **31.15 (+)** | **>40 (-)** | **>40 (-)** | **>40 (-)** |
| Week 9 | **NA (-)** | **NA (-)** | **NA (-)** | **>40 (-)** | **>40 (-)** | **>40 (-)** |

**Case 2**

| **Weeks from COVID-19 VST** | **Nasopharyngeal swab SARS-CoV-2 virus Real-time PCR** | | | **Peripheral blood SARS-CoV-2 virus Real-time PCR** | | |
| --- | --- | --- | --- | --- | --- | --- |
| **COVID-19 genes PCR Ct number, interpretation** | **E** | **Rdrp/S** | **N** | **E** | **Rdrp/S** | **N** |
| Week -6 | **+ NS** | | | **ND** | | |
| Week -4 | **ND** | | | **31.6 (+)** | **32.36 (+)** | **28.81 (+)** |
| Week -3 | **ND** | | | **33.05 (+)** | **34.62 (+)** | **31.08 (+)** |
| Week -2 | **+ NS** | | | **>40 (-)** | **>40 (-)** | **35.46 (+)** |
| Week -1 | **highly + NS** | | | **>40 (-)** | **>40 (-)** | **38.14 (+)** |
| Screening | **25.25 (+)** | **25.91 (+)** | **22.91 (+)** | **>40 (-)** | **>40 (-)** | **39.46 (+)** |
| Week 1 | **33.22 (+)** | **33.5 (+)** | **31.73 (+)** | **>40 (-)** | **>40 (-)** | **>40 (-)** |
| Week 2 | **+ NS** | | | **>40 (-)** | **>40 (-)** | **>40 (-)** |
| Week 2 | **+ NS** | | | **>40 (-)** | **>40 (-)** | **>40 (-)** |
| Week 3 | **ND** | | | **>40 (-)** | **>40 (-)** | **>40 (-)** |
| Week 3 | **-** | | | **>40 (-)** | **>40 (-)** | **>40 (-)** |
| Week 8 | **-** | | | **ND** | | |

**Case 3**

| **Weeks from COVID-19 VST** | **Nasopharyngeal swab SARS-CoV-2 virus Real-time PCR** | | | **Peripheral blood SARS-CoV-2 virus Real-time PCR** | | |
| --- | --- | --- | --- | --- | --- | --- |
| **COVID-19 genes PCR Ct number, interpretation** | **E** | **Rdrp/S** | **N** | **E** | **Rdrp/S** | **N** |
| Week -11 | **29.68 (+)** | **29.62 (+)** | **29.85 (+)** | **>40 (-)** | **>40 (-)** | **>40 (-)** |
| Week -10 | **22.83 (+)** | **22.4 (+)** | **23.26 (+)** | **ND** | | |
| Week -9 | **19.81 (+)** | **18.69 (+)** | **19.08 (+)** | **ND** | | |
| Week -6 | **21.03 (+)** | **20.11 (+)** | **20.07 (+)** | **+ NS** | | |
| Week -3 | **21.72 (+)** | **20.47 (+)** | **19.44 (+)** | **>40 (-)** | **>40 (-)** | **>40 (-)** |
| Week -2 | **24.12 (+)** | **23.39 (+)** | **22.48 (+)** | **>40 (-)** | **>40 (-)** | **>40 (-)** |
| Screening | **20.17 (+)** | **19.59 (+)** | **20.53 (+)** | **>40 (-)** | **>40 (-)** | **>40 (-)** |
| Week 1 | **26.17 (+)** | **25.62 (+)** | **25.19 (+)** | **>40 (-)** | **>40 (-)** | **>40 (-)** |
| Week 1 | **27.09 (+)** | **ND** | | **>40 (-)** | **>40 (-)** | **>40 (-)** |
| Week 2 | **+ NS** | | | **>40 (-)** | **>40 (-)** | **>40 (-)** |
| Week 3 | **36.74 (+)** | **ND** | | **>40 (-)** | **>40 (-)** | **>40 (-)** |
| Week 4 | **>40 (-)** | **>40 (-)** | **>40 (-)** | **>40 (-)** | **>40 (-)** | **>40 (-)** |

Note: yellow background: negative, Ct >40 ; green background: positive, Ct<40; blue background: VST administration.

Abbreviations: VST: virus specific T-cell; PCR: polymerase chain reaction; Ct: cycle threshold; E, Rdrp/S and N: SARS-CoV-2 genes;

NA: not applicable; ND: not done; NS: not specified.
