# Supplementary material for: Effective virus-specific T-cell therapy for high-risk SARS-CoV-2 infections in hematopoietic stem cell transplant recipients: initial case studies and literature review
Source: GeroScience. 2023 Jul 6;46(1):1083–106. doi: 10.1007/s11357-023-00858-7 (PMC10828167; doi:10.1007/s11357-023-00858-7)
Supplement: Supplementary file 5 — Supplementary file5 (DOCX 22 KB) [file 11357_2023_858_MOESM5_ESM.docx]

**Supplementary Table 5.: Changes of multi-cytokine and chemokine levels at screening and after SARS-CoV-2 VST treatment.**

| **Patient** | **Case 1** | | | | **Case 2** | | | | **Case 3** | | |
| --- | --- | --- | --- | --- | --- | --- | --- | --- | --- | --- | --- |
| **Time from COVID-19 VST** | **Screening** | **Week 1** | **Week 2** | **Week 3** | **Screening** | **Week 1** | **Week 2** | **Week 3** | **Screening** | **Week 1** | **Week 2** |
| **Cytokine value (pg/ml)** | | | | | | | | | | | |
| **IFNα2** (208-432) | **46.82** | **17.38** | **33.19** | **<8.0** | **<8.0** | **14.33** | **<8.0** | **<8.0** | **<8.0** | **28.25** | **<8.0** |
| **IFNγ** (22-45) | **18.81** | **7.76** | **17.65** | **<1.3** | **<1.3** | **3.33** | **<1.3** | **<1.3** | **16.98** | **73.25** | **17.82** |
| **IL-1α** (140-291) | **29.80** | **<4.8** | **7.45** | **<4.8** | **7.2** | **<4.8** | **<4.8** | **<4.8** | **<4.8** | **42.88** | **<4.8** |
| **IL-1β** (45-94) | **39.12** | **14.07** | **19.20** | **8.06** | **2.94** | **5.80** | **<1.6** | **<1.6** | **2.94** | **11.11** | **4.87** |
| **IL-2** (18-38) | **2.66** | **0.78** | **1.27** | **<0.64** | **1.21** | **2.12** | **1.21** | **<0.64** | **1.09** | **12.86** | **3.15** |
| **IL-4** (19-40) | **10.75** | **2.56** | **3.94** | **1.45** | **1.64** | **2.40** | **2.08** | **<0.64** | **<0.64** | **0.80** | **<0.64** |
| **IL-5** (21-44) | **29.02** | **9.38** | **10.97** | **4.57** | **3.17** | **9.79** | **21.75** | **6.54** | **2.55** | **21.46** | **16.97** |
| **IL-6** (20-42) | **60.64** | **25.94** | **69.98** | **3.48** | **65.81** | **23.48** | **14.64** | **5.21** | **3.48** | **74.67** | **11.90** |
| **IL-8** (21-44) | **92.49** | **46.16** | **44.60** | **18.43** | **29.54** | **51.79** | **62.19** | **3.95** | **21.67** | **23.39** | **28.88** |
| **IL-10** (87-181) | **68.65** | **18.32** | **25.39** | **4.90** | **199.95** | **183.70** | **44.53** | **<2.6** | **3.87** | **623.18** | **239.53** |
| **IL-12 (p70)** (103-214) | **4.65** | **<3.0** | **3.07** | **<3.0** | **<3.0** | **<3.0** | **<3.0** | **<3.0** | **3.54** | **4.65** | **<3.0** |
| **IL-13** (197-409) | **86.26** | **21.08** | **23.14** | **<3.0** | **<3.0** | **<3.0** | **<3.0** | **<3.0** | **<3.0** | **32.67** | **<3.0** |
| **IL-15** (104-216) | **40.14** | **22.13** | **37.71** | **18.51** | **38.79** | **28.74** | **16.54** | **7.63** | **12.01** | **31.74** | **17.11** |
| **IL-17A** (40-82) | **14.63** | **3.60** | **7.37** | **<1.3** | **<1.3** | **4.16** | **<1.3** | **<1.3** | **1.80** | **6.85** | **3.60** |
| **IP-10/ CXCL10** (67-139) | **1995.93** | **876.01** | **1767.99** | **859.58** | **696.76** | **1125.65** | **1513.76** | **615.98** | **890.44** | **1886.76** | **1376.34** |
| **MCP-1/ CCL2** (101-210) | **2309.59** | **914.15** | **1331.50** | **350.03** | **1637.01** | **1618.34** | **1260.95** | **679.32** | **1099.26** | **3563.54** | **2033.26** |
| **MIP-1α/CCL3** (74-153) | **81.06** | **37.75** | **42.75** | **23.27** | **35.40** | **36.59** | **21.68** | **6.93** | **<3.0** | **14.44** | **11.09** |
| **RANTES/CCL-5** (40-84) | **5764.25** | **2450.40** | **4410.17** | **1183.11** | **5189.95** | **5984.34** | **4165.01** | **4649.02** | **5406.67** | **8442.85** | **5721.83** |
| **TNFα** (123-256) | **48.21** | **20.54** | **24.89** | **7.06** | **33.74** | **38.24** | **32.41** | **12.78** | **31.88** | **386.51** | **250.56** |
| **TNFβ** (53-111) | **2.65** | **<1.6** | **<1.6** | **<1.6** | **3.36** | **5.01** | **2.88** | **<1.6** | **<1.6** | **3.59** | **<1.6** |

Note: no background: decreased value; blue background: normal value; pink background: >1-<10x upper normal value; purple background: >10-<100x upper normal value; red background: >100x upper normal value.

Abbreviations: VST: virus specific T-cell; IFN: interferon; IL: interleukin; RANTES: regulated upon activation. normal T-cell expressed and secreted; MCP-1: monocyte chemoattractant protein-1; IP-10: interferonγ-induced protein 10 kDa; TNF: tumor necrosis factor.
